# Effect of *Lactobacillus fermentum* ZS40 on the NF-κB signaling pathway in an azomethane-dextran sulfate sodium-induced colon cancer mouse model

**DOI:** 10.3389/fmicb.2022.953905

**Published:** 2022-09-26

**Authors:** Jia Liu, Shuaiqi Wang, Ruokun Yi, Xingyao Long, Xin Zhao

**Affiliations:** ^1^Collaborative Innovation Center for Child Nutrition and Health Development, Chongqing Engineering Research Center of Functional Food, Chongqing Engineering Laboratory for Research and Development of Functional Food, Chongqing University of Education, Chongqing, China; ^2^Gastrointestinal Tumor Center, Chongqing University Cancer Hospital, Chongqing, China

**Keywords:** NF-κB signaling pathway, AOM-DSS, colon cancer, *Lactobacillus fermentum*, inflammation-related genes

## Abstract

The occurrence of intestinal diseases such as colon cancer is closely related to the intestinal flora. *Lactobacillus fermentum* is a gut probiotic that plays an important role in chronic intestinal inflammation and colon cancer. In the current study, we investigated the effect of *Lactobacillus fermentum* ZS40 on NF-κB signaling pathway of azomethane-dextran sulfate sodium (AOM-DSS) -induced colon cancer in mice. Animals were divided into control group (NC), AOM-DSS-induced model group (CRC), AOM-DSS plus high-dose *Lactobacillus fermentum* ZS40 (ZS40-H), AOM-DSS plus low-dose *Lactobacillus fermentum* ZS40 (ZS40-L), AOM-DSS plus *Lactobacillus bulgaricus* (BLA), and AOM-DSS plus sulfasalazine (SD)-treated group. Observation of animal physiological activity (body weight and defecation), biochemical measurements, histopathological examination of colon tissue, qPCR to evaluate the expression of inflammation-related genes, immunohistochemical analysis of CD34 and CD117, and Western blot analysis of NF-κB signaling pathway were performed. Compared with the CRC group, the ZS40-H, ZS40-L, BLA, and SD groups had decreased levels of colon cancer marker proteins CD34 and CD117, and the number of abnormal colonic lesions observed by colon histology decreased, while the ZS40-H group showed excellent results. In addition, all probiotic interventions showed weight loss effects. The expression of inflammatory stimulators TNF-α and IL-1β in the probiotic treatment group decreased; the expression of key proteins IκBα and p65 in the NF-κB signaling pathway also decreased, resulting in a decrease in the expression of the target protein Cox-2. Therefore, administration of *Lactobacillus fermentum* ZS40 as a probiotic can alleviate intestinal inflammation and prevent colon cancer in mice.

## Introduction

With high morbidity and mortality, colon cancer is a malignant disease that threatens both human health and life (Arnold, 2018). Colon cancer ranks third in the incidence of cancer diseases among both men and women, globally (Tang and Evans, 2021). In China, the number of new cases of colon cancer each year is as high as 400,000, and there are about 10 million new cases worldwide annually (Jia et al., 2022). There may be no symptoms in the early stages of colon cancer. In the middle and late stages, the disease may manifest as abdominal distension and indigestion, followed by changes in bowel habits, abdominal pain, or blood in the stool (Pacal et al., 2020). The methods for early detection of colon cancer are imprecise because the symptoms in the early stages of disease are not obvious, which leads to the late detection of colon cancer (Birgisson et al., 2021). At present, some patients with colon cancer can be treated with surgery, but the risks involved in surgery are high and the adverse postoperative effects can be considerable (Wahab et al., 2021).

Inflammation is the body’s defense response to injury, but uncontrolled inflammation is often closely associated with cancer development and metastasis (Schmitt and Greten, 2021). Sometimes, the chronic inflammation that causes cancer stems from a disease characterized by inflammation. For example, the inflammatory diseases colitis, pancreatitis, and hepatitis are associated with an increased risk of colon cancer, pancreatic cancer, and liver cancer, respectively. Clinical investigations have shown that many cancer patients have a history of chronic inflammatory diseases (Beckmann et al., 2019). Numerous studies have confirmed that inflammation may also disrupt the balance of intestinal flora and induce intestinal diseases (Jackson and Theiss, 2020). In chronic inflammation, cytokines and chemokines produced by inflammatory cells can spread the focal inflammatory response to surrounding tissues through the NF-κB signaling pathway (Zhao et al., 2021). After induction by pro-inflammatory factors and tumor necrosis factors, the NF-κB signaling pathway regulates the expression of various genes such as interleukin-related genes and apoptotic factors downstream. This process also increases the immune evasion ability of precancerous cells. The inflammatory process produces molecules called cytokines that stimulate the growth of blood vessels that bring oxygen and nutrients to the tumor, and the process can also produce molecules called free radicals that further damage DNA. These inflammatory side effects may help maintain and promote cancer growth. Therefore, chronic inflammation is the initiating factor of tumorigenesis, and NF-κB plays an important role in the occurrence and development of inflammatory tumors.

The human colon is an important metabolic organ with a complex intestinal flora structure (Zhao Y. L. et al., 2018). The number of viable bacterial cells per gram of intestinal content is far greater than 10^11^—and can even reach 10^14^ (Li C. et al., 2019). In general, the intestinal flora can protect the colon, but when the number of florae is reduced by more than 50%, the ability to protect the colon from carcinogens is lost (Zhou et al., 2019). Gut microbes regulate gut health by releasing metabolites, and dysbiosis is a hallmark of colorectal cancer (CRC), leading to inflammation, tumor growth, and response to therapy (Bell et al., 2022). Studies conducted at the beginning of the twentieth century found that the longevity of people from Bulgaria was related to their long-term use of fermented dairy products (Salvetti and O’Toole, 2017). Since then, a large amount of research data has shown that the intake of fermented dairy products is beneficial to human health (Warren et al., 2018).

*Lactobacillus fermentum* ZS40 (China General Microbiological Culture Collection Center, CGMCC No.: 18226) is an active strain isolated from the traditionally fermented yogurt in Zhaosu County, Xinjiang, by the Chongqing Collaborative Innovation Center for Functional Food. The preliminary activity test results have shown that the ability of *Lactobacillus fermentum* ZS40 to tolerate artificial gastric juice reached 79.3%. In this study, a mouse colon cancer model induced by azoxymethane-dextran sulfate sodium (AOM-DSS) was used to explore the influence of the NF-κB signaling pathway in the occurrence and development of colon cancer and the ameliorative effect of *Lactobacillus fermentum* ZS40.

## Materials and methods

### Strain culture

*Lactobacillus fermentum* ZS40 and *Lactobacillus bulgaricus* strains were inoculated into Man, Rogosa (MRS) liquid medium at an inoculum of 2%. and was incubated in a constant temperature water shaker at 37°C at 100 rpm for 24 h. The solution was then centrifuged at 12,000 rpm for 10 min and the supernatant discarded. The bacterial pellet was then resuspended in 0.9% saline. The purity of the bacterial solution was checked with Gram stain microscopy. Using the gradient dilution method, the *Lactobacillus fermentum* ZS40 bacterial solution was diluted to 10^11^ and 10^9^ colony-forming units (CFU), and *Lactobacillus bulgaricus* was diluted to 10^11^ CFU for use.

### *In vitro* resistance of probiotic

The MRS-THIO medium with porcine bile salt concentrations of 0, 1, 2, and 3 g/L was prepared, the activated probiotic were inoculated into the MRS-THIO medium at a volume of 2%, and the blank medium with a bile salt concentration of 0 was used as a control. After culturing at 37°C and 100 rpm for 24 h, the absorbance A at OD600nm was measured, and the tolerance of probiotic to bile salts was calculated according to formula 1.


(1) Bilesalttolerance=A1/A0×100%

A1: OD600 nm of bile salt-containing medium; A0: OD600 nm of blank medium.

Artificial gastric juice prepared with 0.2% NaCl and 0.35% pepsin was used, pH3.0 was adjusted to use 1 mol/L HCl, and the bacteria were filtered through a 0.22 μm filter. The activated probiotic and artificial gastric juice were mixed in a volume ratio of 1:9. They were incubated at 37°C and 100 rpm. The culture medium after 0 h and 3 h was taken and diluted 10 times with 0.9% NaCl. The diluted solution was spread on MRS solid medium plate, and cultured at 37°C for 48h. The survival rate of probiotic was calculated according to formula 2.


(2) $$\begin{array}{l} \mathrm{Survival}\;\mathrm{rate}=3h\mathrm{number}\;\mathrm{of}\;\mathrm{viable}\;\mathrm{probiotic}\\ \;\;\;\;\;\;\;\;\;\;\;\;\;\;\;\;\;\;\;\;\;\;\;\;\;\;\;\;\;\;\;\;\;\;\;/0h\mathrm{number}\;\mathrm{of}\;\mathrm{viable}\;\mathrm{probiotic}\times 100\% \end{array}$$

### Experimental animals

**Figure 1:**
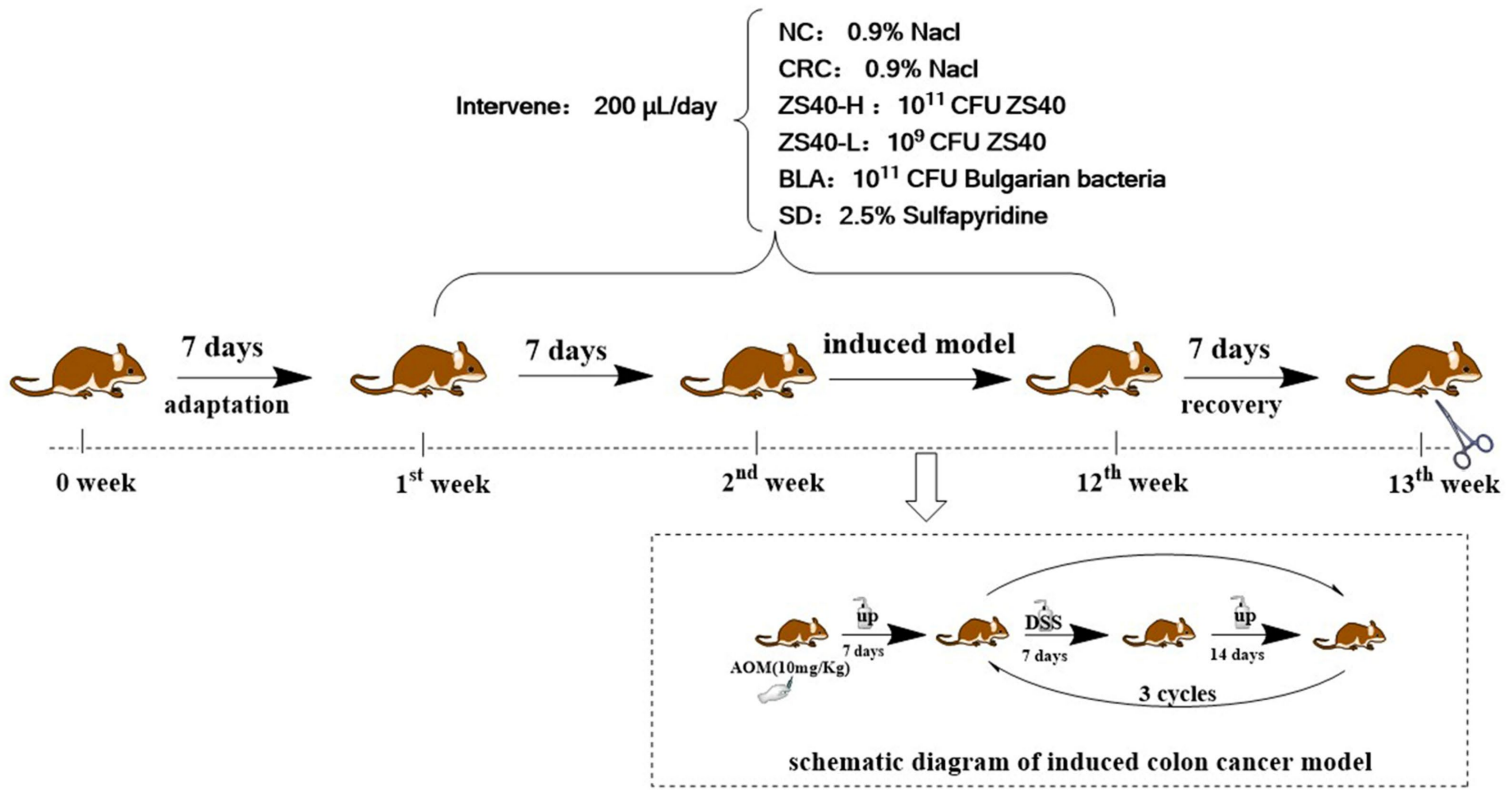
Schematic diagram of experiment cycle.

Male C57BL/6 J mice (4 weeks old, 16–18 g) were purchased from Chongqing Medical University. Mice were housed at 25°C under a 12-h light/dark cycle and provided with standard rodent chow (ENSIWEIER Biotechnology Co., Ltd., Chongqing, China) and purified water. The entire trial period was 12 weeks. The 1st week was the adaptive culture cycle. All experimental animals were provided with standard feed and purified water. After the adaptation period, the experimental animals were randomly divided into Normal group (NC); Colorectal cancer control group (CRC); Probiotic intervention group: high-dose ZS40 bacterial solution (ZS40-H), low-dose ZS40 bacterial solution (ZS40-L) and Bulgarian bacteria bacterial solution (BLA), and Drug control group: sulfasalazine solution (SD). The 2nd to the 11th week were model induction cycle, NC and CRC were given 0.2 ml of 0.9% Nacl/Day; ZS40-H, ZS40-L, and BLA group were administered by gavage with 0.2 ml of 10^11^ CFU, 10^9^ CFU, and 10^11^ CFU bacterial solution, respectively, as the number of viable bacteria ingested per day; SD was given 0.2 ml of 2.5% sulfasalazine solution by intragastric administration per day. In addition to the above continuous treatment operations, the following treatments are also required. On the first day of the 2nd week, all groups except NC were intraperitoneally injected with AOM reagent at a dose of 10 mg/kg, and purified water was provided during the week. From 3th to 5th week, drinking conditions of 2.5% DSS solution for 7 days and purified water for 14 days were provided. These drinking conditions were repeated 3 times (3th–11th week). The 12th week was the recovery week, and all treatments of all experimental animals were stopped and were provided with standard feed and purified water (Figure 1). After all experiments (13th week), all animals blood was collected by orbital draw method and centrifuged at 9,500*g* at 4°C in a refrigerated high-speed centrifuge (iCEN-24R, Hangzhou Aosheng Instrument Co., Ltd., Zhejiang, China) to obtain serum, and dissected and collected tissues. The colon were quickly removed, and photographed. The intercepted part of the sample was immersed in the fixative at 4°C and sent to the biological company for staining. The remaining colon samples were immediately frozen in liquid nitrogen and stored at −80°C for further analysis. Animal experiments were approved by the Ethics Committee of Chongqing Collaborative Innovation Center for Functional Food (IACUC Number: 202106009B).

### Colon morphology observation

The sacrificed mice were fixed on the dissecting board, the intestinal tissue of the mice was removed, the connection between the cecum and the small intestine was carefully cut, the connection between the colon and the anus was cut off, and the colon tissue was obtained and weighed. The colon was carefully unfolded. Been careful not to artificially lengthen the colon. Be careful not to artificially increase the length of the colon. The 0-scale position was aligned with the junction of the colon and the cecum.

### Colon pathological observation

Colon tissue samples from each animal were pathologically examined. These tissue samples were fixed in a tissue fixative (Sevier Biotechnology Co., Ltd., Chongqing, China) at 4°C (Collected in 2.1). Within 24 h, the fixed tissue samples were sent to Chongqing Sevier Biological Company to prepare paraffin sections and undergo hematoxylin and eosin (H&E) staining. Additionally, the obtained tissue sections were sent to Chongqing Servicebio Biological Company again for CD34 (GB121693, Servicebio) and CD117 (GB11073-2, Servicebio) immunohistochemical staining. The stained samples were viewed with an upright microscope (BX43F; Olympus, Tokyo, Japan). And the severity of intestinal pathology was double-blindly scored, pathology score ≥ 2, regarded as intestinal injury.

### Serum enzyme-linked immunosorbent assay (Elisa)

To prepare serum samples, the collected blood *via* retro-orbital sampling was allowed to stand at 4°C for 1 h and centrifuged at 9,500*g* for 15 min at 4°C (Collected in 2.1). A commercial ELISA kit (mlibio, Shanghai, China) was then used to measure levels of interleukin 1β (IL-1β), interleukin 8 (IL-8), tumor necrosis factor-α (TNF-α), macrophage inflammatory protein 1β (MIP-1β), and vascular endothelial cell adhesion molecule 1 (VCAM-1).

### Real-time fluorescence quantitative PCR

The mRNA expressions of IL-1β, TNF-α, nuclear factor kappa-B (NF-κB, p65), TNF receptor-associated factor 1/2/6 (TRAF1/2/6), nuclear factor inhibitor protein (IκBα), IκB kinase (IKKα/β), B-cell lymphoma/leukemia (Bcl-XL, Bcl-2), and Cyclooxygenase (Cox-2) were measured using real-time fluorescence quantitative PCR (RT-qPCR). The total RNA was extracted and cDNA reverse transcription was performed, according to instructions of the RNA extraction kit (BaiMaiKe Technologies, Beijing, China) and cDNA reverse transcription kit (Yeasen Technologies, Shanghai, China), respectively. A microspectrophotometer (Nano-300; ALLSHENG, Zhejiang, China) was used for quantification by measuring the absorbance at 260 nm. Then, RT-qPCR was performed using an RT-qPCR instrument (StepOne Plus, ABI, United States), normalized with actin. The final RT-qPCR product expression was calculated using the 2^−△△Ct^ method. Each sample was replicated 4 times and data were statistically analyzed using Prism 7.0 software.

### Western blot analysis

Colon samples were homogenized using a protein extraction kit, in which the phenylmethanesulfonyl fluoride, protease inhibitors, and phosphatase inhibitors were mixed (Solarbio Life Sciences, Beijing, China), and then centrifuged at 12,000*g* for 20 min at 4°C. The protein concentration was determined using a bicinchoninic acid protein determination kit (Yeasen Technologies, Shanghai, China). For Western blot analysis, 50 μg of protein extract was separated with 10% NuPAGE (NP0302BOX, Invitrogen) and then transferred to a polyvinylidene fluoride (PVDF) membrane. This was sealed with 5% skim milk for 1 h at 28°C, using anti-p65 (51-0500, Invitrogen), anti-IL-1β (MM425B, Invitrogen), anti-TNF-α (AMC3012, Invitrogen), anti-IκBα (MA5-16152, Invitrogen), anti-Cox-2 (PA5-17614, Invitrogen), and anti-β-actin (MA1-140, Invitrogen); incubated in the PVDF membrane; washed five times with 1× TBST; and then combined with Horseradish peroxidase (HRP) secondary antibodies (A32723, Invitrogen). Next, the mixture was incubated for 1 h at room temperature and the PVDF membrane was washed five times with 1× TBST. Antibody binding was observed using enhanced chemiluminescence (Solarbio Life Sciences, Beijing, China). Finally, ImageJ software (U.S. National Institutes of Health, Bethesda, MD, United States) was used to quantify protein expression.

### Data analysis

The data were expressed as mean ± standard deviation (SD). GraphPad 7.0 (GraphPad Software, San Diego, CA, United States) and IBM SPSS 21.0 (IBM Corp., Armonk, NY, United States) statistical software packages were used for analysis. We used one-way analysis of variance and Duncan’s multiple range test to evaluate the difference between the mean values in each group. A value *p* < 0.05 was considered statistically significant.

## Results

### Viability of probiotics

Both *Lactobacillus fermentum* ZS40 and *Lactobacillus bulgaricus* could grow in an MRS medium containing different concentrations of bile salts, but the growth efficiency varies greatly under different concentrations. In 1 g/L MRS-THIO medium, the growth efficiency of *Lactobacillus fermentum* ZS40 and *Lactobacillus bulgaricus* was over 50%, and the growth efficiency of *Lactobacillus fermentum* ZS40 reached 79%; with the increase of bile salt concentration, the growth efficiency of *Lactobacillus fermentum* ZS40 and *Lactobacillus bulgaricus* was inhibited. Under the condition of concentration of 2 g/L, the growth efficiency of *Lactobacillus fermentum ZS40* reached 43%; under the condition of concentration of 3 g/L, the growth efficiency of *Lactobacillus fermentum ZS40* was 28%.

There was a difference in the survival rates of *Lactobacillus fermentum* ZS40 and *Lactobacillus bulgaricus* in artificial gastric juice at pH3.0. The viable counts of *Lactobacillus fermentum* ZS40 and *Lactobacillus bulgaricus* in artificial gastric juice were 6.3 × 10^7^ CFU/ml and 4.8 × 10^7^ CFU/ml after 0 h; the viable counts after 3 h were 4.27 × 10^6^ CFU/ml and 2.88 × 10^6^ CFU/ml; the survival rates of *Lactobacillus fermentum* ZS40 and *Lactobacillus bulgaricus* in the artificial gastric juice of pH3.0 within 3 h were, respectively, 6.7 and 6%.

### Effect of probiotic samples on body weight

**Figure 2:**
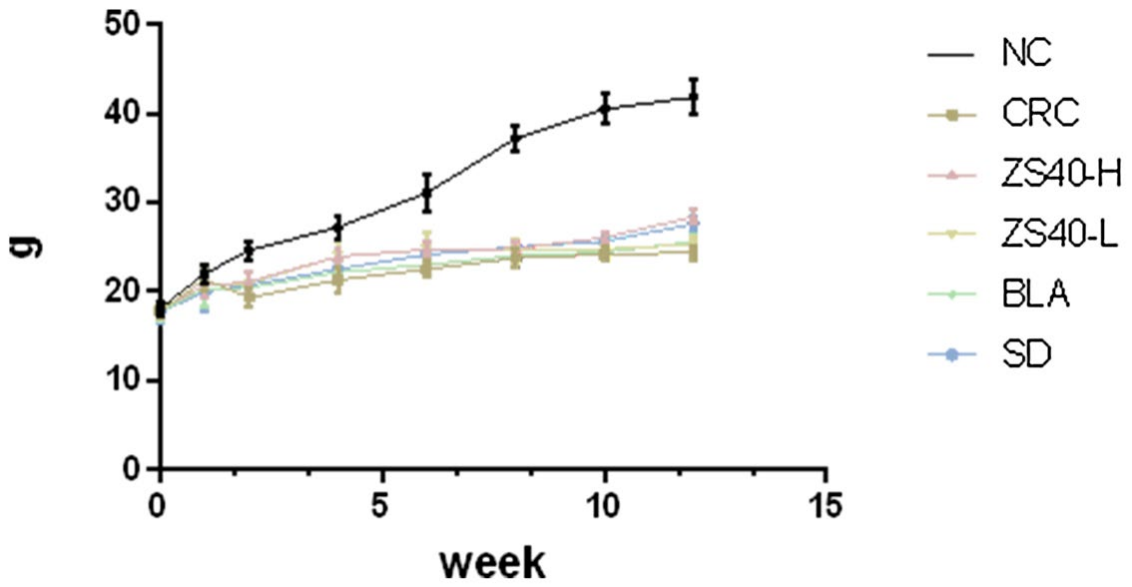
Body weight of mice. NC, normal untreated mice; CRC, colon cancer model mice induced with AOM-DSS; ZS40-H, mice treated with high-dose *Lactobacillus fermentum* ZS40 (10^11^ CFU); ZS40-L, mice treated with low-dose *Lactobacillus fermentum* ZS40 (10^9^ CFU); BLA, mice treated with Bulgarian strain (10^11^ CFU); SD, mice treated with sulfasalazine (25%).

During the experiment, the body weight changes of the experimental mice were recorded. As shown in Figure 2, from the 2th experimental week after the injection of AOM, compared with the normal group, the body weight of the mice in the CRC group showed a significant decline. In the later stage of the experiment, the body weight of the mice in the CRC group kept decreasing under the action of DSS. Although the body weight of the mice in the intervention group of *Lactobacillus fermentum* ZS40-H was affected by AOM-DSS, the body weight was upregulated, showing differences from CRC mice. The same results were also shown in the ZS40-L, BLA, and SD groups. The body weight of mice in these intervention groups all showed a certain degree of upregulation.

### Effect of probiotic samples on colon morphology

**Figure 3:**
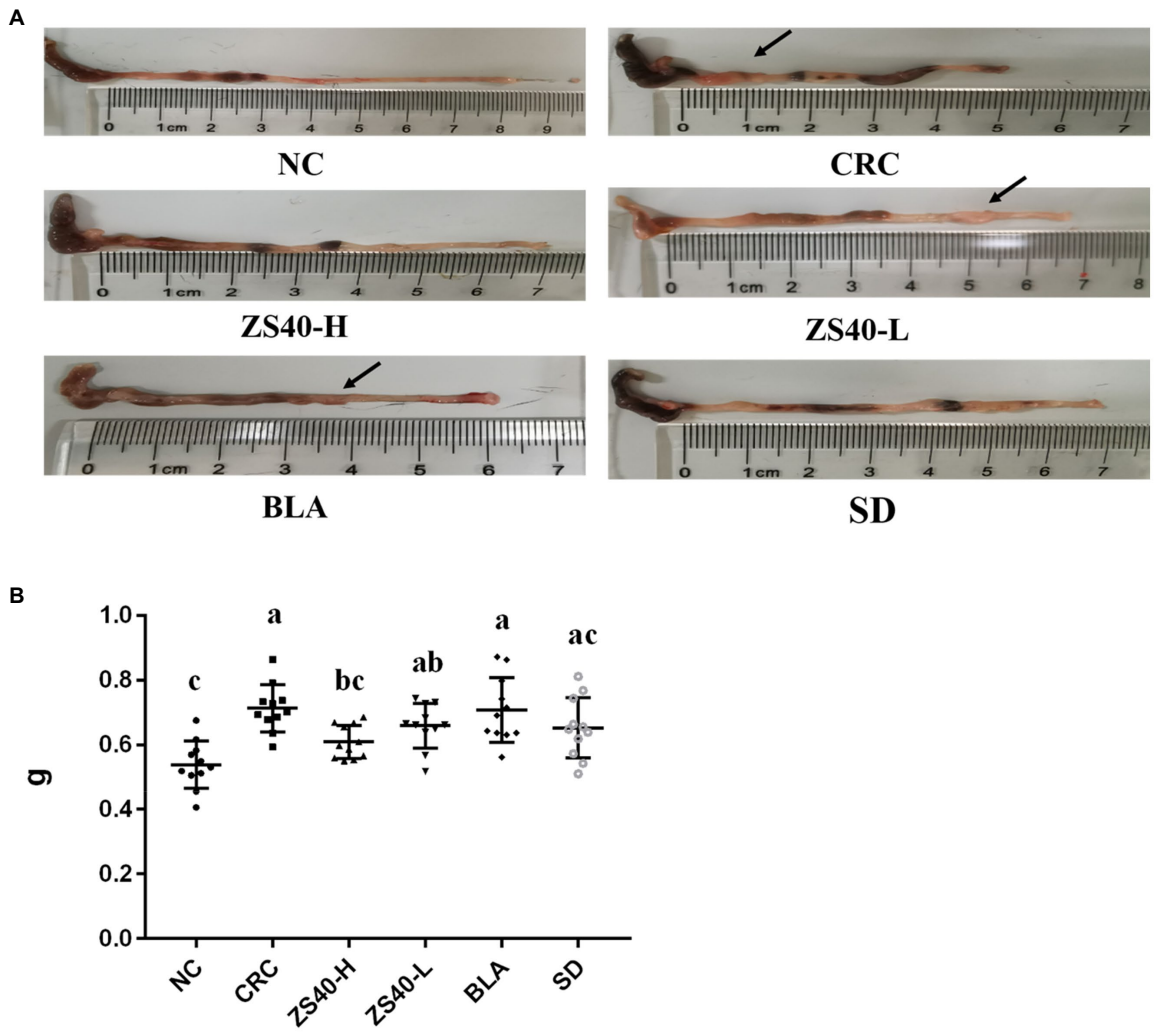
Anatomy of mice colon **(A)**: Colon length; **(B)** Colon weight. ^a–c^Mean values with different letters over the same column are significantly different (*p* < 0.05) according to Duncan’s multiple range test. NC, normal untreated mice; CRC, colon cancer model mice induced with AOM-DSS; ZS40-H, mice treated with high-dose *Lactobacillus fermentum* ZS40 (10^11^ CFU); ZS40-L, mice treated with low-dose *Lactobacillus fermentum* ZS40 (10^9^ CFU); BLA, mice treated with Bulgarian strain (10^11^ CFU); SD, mice treated with sulfasalazine (25%).

As shown in Figure 3A, the length and morphology of the mice colon were compared after dissection at the end of the experiment, and the colon weight was recorded (as shown in Figure 3B). In the anatomical image, we can see that compared with NC, owing to the large amount of inflammation produced during the process of inducing colon cancer, the length of the colon in CRC mice was significantly shortened (NC:8.3 cm; CRC:4.9 cm), the intestine was filled with pus-like material, colon weight increased significantly(NC:0.5370 ± 0.08 g; CRC:0.7245 ± 0.06 g), and colon lumps were found at multiple locations (Where the arrow points). On the contrary, in ZS40-H and SD mice, there was less shortening of the colon (ZS40-H:7.0 cm; SD:6.9 cm), less pus-like material, fewer colon lumps, and the volume of the lumps was also reduced (ZS40-H:0.603 ± 0.05 g; SD:0.6555 ± 0.10 g). Even, no lumps were found in some tissues. Similar situations were found in the ZS40-L and BLA groups (ZS40-L:6.7 cm, 0.6729 ± 0.05 g; BLA:6.0 cm, 0.6926 ± 0.09 g), but the improvement was not more obvious than that in ZS40-H and SD mice. This shows that after the interference of probiotics and drugs, inflammation in the mouse colon was alleviated, and the effects of high-dose ZS40 and drugs were the most significant.

### Effect of probiotics samples on serum indexes

As shown in Table 1, compared with NC mice, the serum levels of IL-1β, IL-8, TNF-α, MIP-1β, and VCAM-1 in the CRC group were significantly increased. Compared with CRC, ZS40-H treatment reduced the serum levels of IL-1β, IL-8, TNF-α, MIP-1β, and VCAM-1 in CRC mice. Moreover, compared with the CRC group, mice in the ZS40-L and BLA treatment groups and the SD control group had effectively reduced levels of IL-1β, IL-8, TNF-α, MIP-1β, and VCAM-1; SD showed a better effect in reducing the levels of inflammatory factors (*p* < 0.05). This indicates that *Lactobacillus fermentum* ZS40 could reduce the levels of inflammatory factors and vascular cell adhesion factors in the serum of C57 mice induced by AOM-DSS.

**Table 1:** Levels of inflammatory indexes in mouse serum samples.

	IL-1β (pg/ml)	IL-8 (pg/ml)	TNF-α (pg/ml)	MIP-1 (pg/ml)	VCAM-1 (pg/ml)
NC	22.675 ± 1.32^bc^	24.274 ± 1.46^c^	162.356 ± 15.04^c^	5.209 ± 0.37^c^	92.830 ± 6.75^c^
CRC	30.697 ± 2.95^a^	34.750 ± 2.78^a^	257.238 ± 19.32^a^	8.113 ± 0.50^a^	143.874 ± 15.11^a^
ZS40-H	24.432 ± 1.08^c^	25.436 ± 1.22^c^	202.718 ± 15.76^b^	5.665 ± 0.69^bc^	94.332 ± 7.11^c^
ZS40-L	26.069 ± 1.22^b^	26.661 ± 1.71^bc^	212.658 ± 13.28^b^	7.618 ± 0.38^a^	129.738 ± 14.81^b^
BLA	26.639 ± 1.46^b^	27.681 ± 1.36^b^	209.094 ± 17.04^b^	7.678 ± 0.62^a^	119.048 ± 11.73^b^
SD	26.058 ± 1.85^b^	24.650 ± 1.39^c^	193.632 ± 21.78^b^	6.106 ± 0.42^b^	100.738 ± 6.36^c^

Values are mean ± standard deviation (*N* = 10/group).

^a–c^Mean values with different letters over the same column are significantly different (*p* < 0.05) according to Duncan’s multiple range test. NC, normal untreated mice; CRC, colon cancer model mice induced with AOM-DSS; ZS40-H, mice treated with high-dose Lactobacillus fermentum ZS40 (10^11^ CFU); ZS40-L, mice treated with low-dose Lactobacillus fermentum ZS40 (10^9^ CFU); BLA, mice treated with Bulgarian strain (10^11^ CFU); SD, mice treated with sulfasalazine (25%); IL-1β, interleukin 1β; IL-8, interleukin 8; TNF-α, tumor necrosis factor-α; MIP-1β, macrophage inflammatory protein 1β; VCAM-1, vascular endothelial cell adhesion molecule 1.

### Effect of probiotic samples on colon tissue

**Figure 4:**
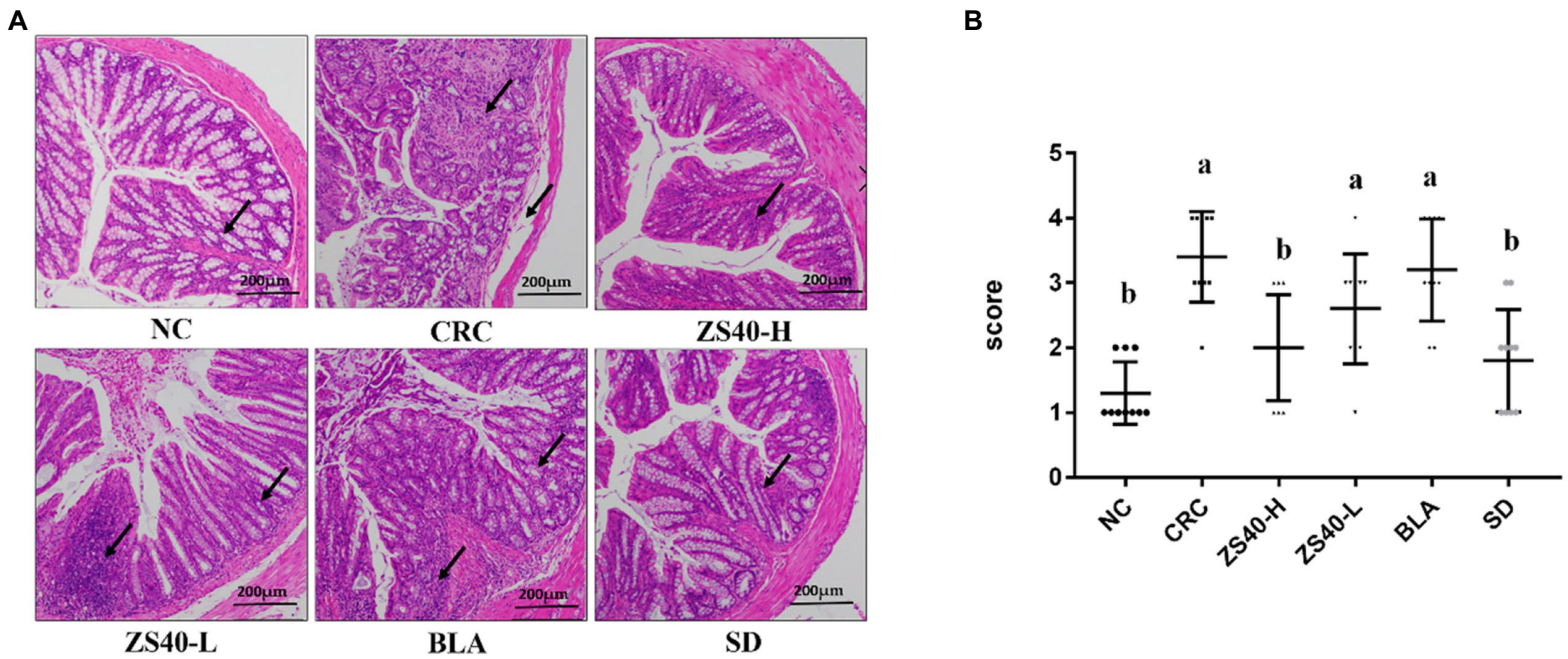
**(A)** Pathological section of mice colon (H&E) Magnification 10×. **(B)** Colon severity pathological scores 0 marks: intact villi and epithelium; 1 marks: slight submucosal or lamina propria swelling and separation; 2 marks: moderate submucosa or lamina propria swelling and separation and plasma cell infiltration; 3 marks: severe submucosal or lamina propria swelling and plasma cell infiltration, local villi atrophy and shedding; 4 marks: intestinal villi disappeared with intestinal wall necrosis Pathology score ≥ 2, regarded as intestinal injury. ^a,b^Mean values with different letters over the same column are significantly different (*p* < 0.05) according to Duncan’s multiple range test. NC, normal untreated mice; CRC, colon cancer model mice induced with AOM-DSS; ZS40-H, mice treated with high-dose *Lactobacillus fermentum* ZS40 (10^11^ CFU); ZS40-L, mice treated with low-dose *Lactobacillus fermentum* ZS40 (10^9^ CFU); BLA, mice treated with Bulgarian strain (10^11^ CFU); SD, mice treated with sulfasalazine (25%) The arrows point to the colonic mucosa and goblet cell structure.

The morphology of colon tissue was observed by analyzing the H and E-stained sections of colon tissue (Figure 4A), and the pathological score of the severity of the colon of the mice (Figure 4B). In the CRC group, epithelial cells fall off in colon tissue, and lymphocytes and plasma cells infiltrate in lamina propria. We observed that the ZS40-H intervention treatment could reduce the accumulation of inflammatory factors in the colon tissue of CRC mice. Also, compared with the CRC group, the ZS40-L and BLA treatment groups and the BD control group had significantly reduced inflammatory infiltration of mouse colon tissues.

### Effect of probiotic samples on expression of tumor markers

**Figure 5:**
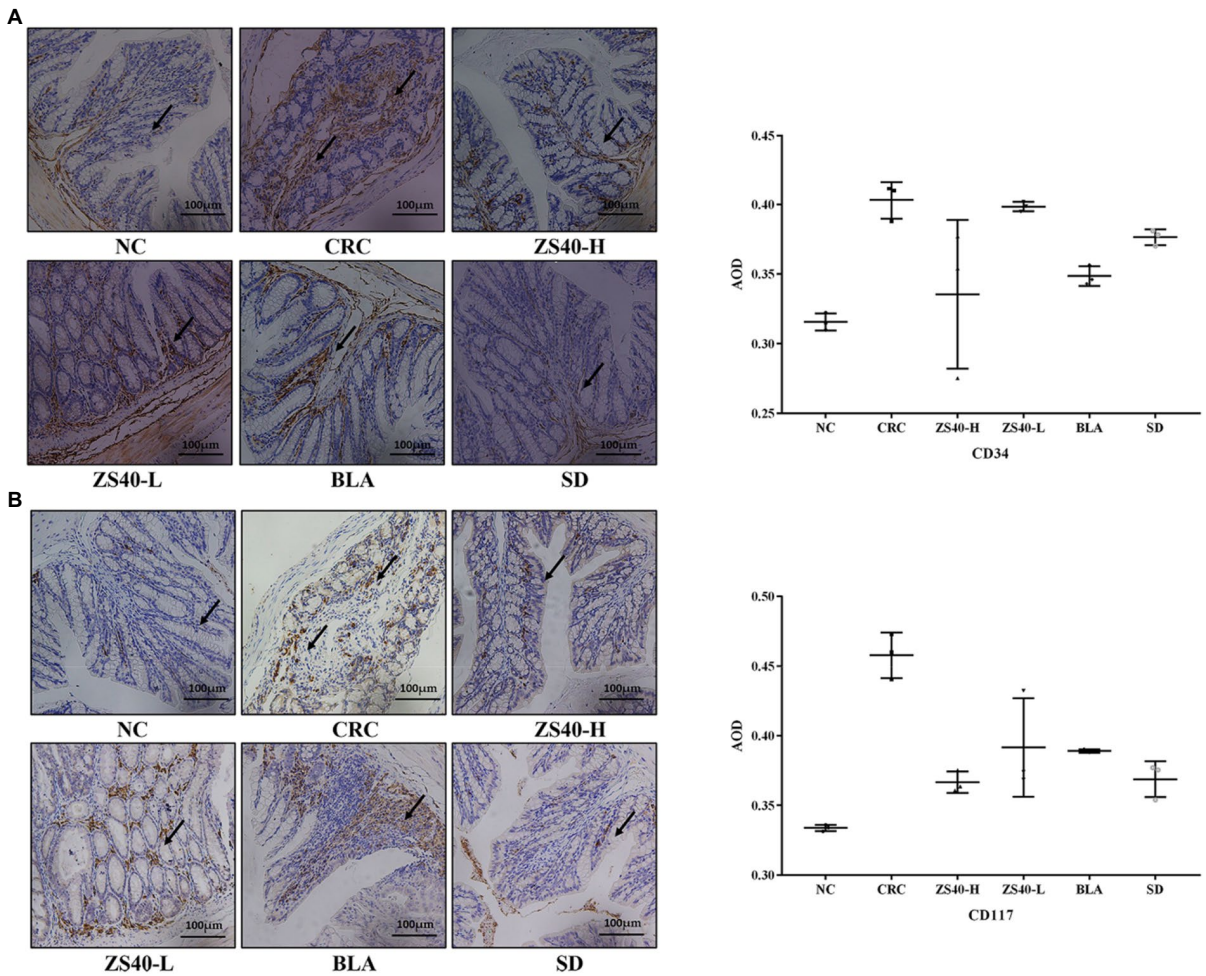
Pathological section of mice colon (immunohistochemical staining) Magnification 20× **(A)**: Indicator CD34; **(B)**: Indicator CD117; NC, normal untreated mice; CRC, colon cancer model mice induced with AOM-DSS; ZS40-H, mice treated with high-dose *Lactobacillus fermentum* ZS40 (10^11^ CFU); ZS40-L, mice treated with low-dose *Lactobacillus fermentum* ZS40 (10^9^ CFU); BLA, mice treated with Bulgarian strain (10^11^ CFU); SD, mice treated with sulfasalazine (25%) The arrows point to the expression of the target protein.

The staining intensities of CD34 and CD117 expression in colon tissue were observed with immunohistochemistry. As shown in Figure 5, compared with the NC group (AOD: CD34: 0.334 ± 0.01; CD117: 0.316 ± 0.01), the expression intensities of CD34 and CD117 were higher in colon tissue samples of the CRC group. In colon lesions, the expression levels of target cells (CD34 and CD117) were increased (AOD: CD34: 0.403 ± 0.01; CD117: 0.458 ± 0.02), the expression aggregation area of the target protein could be clearly observed, and the expression rates of positive results were higher. Compared with CRC, after intervention with ZS40-H, ZS40-L, BLA, and SD, the positive expression rate decreased (AOD: CD34 ZS40-H: 0.335 ± 0.05, ZS40-L: 0.398 ± 0.01, BLA: 0.349 ± 0.01, SD: 0.376 ± 0.01; CD117 ZS40-H: 0.367 ± 0.01, ZS40-L: 0.392 ± 0.04, BLA: 0.389 ± 0.01, SD: 0.369 ± 0.01). Among these, the ZS40-H and SD groups were more obvious.

### Effect of probiotic samples on mRNA

**Figure 6:**
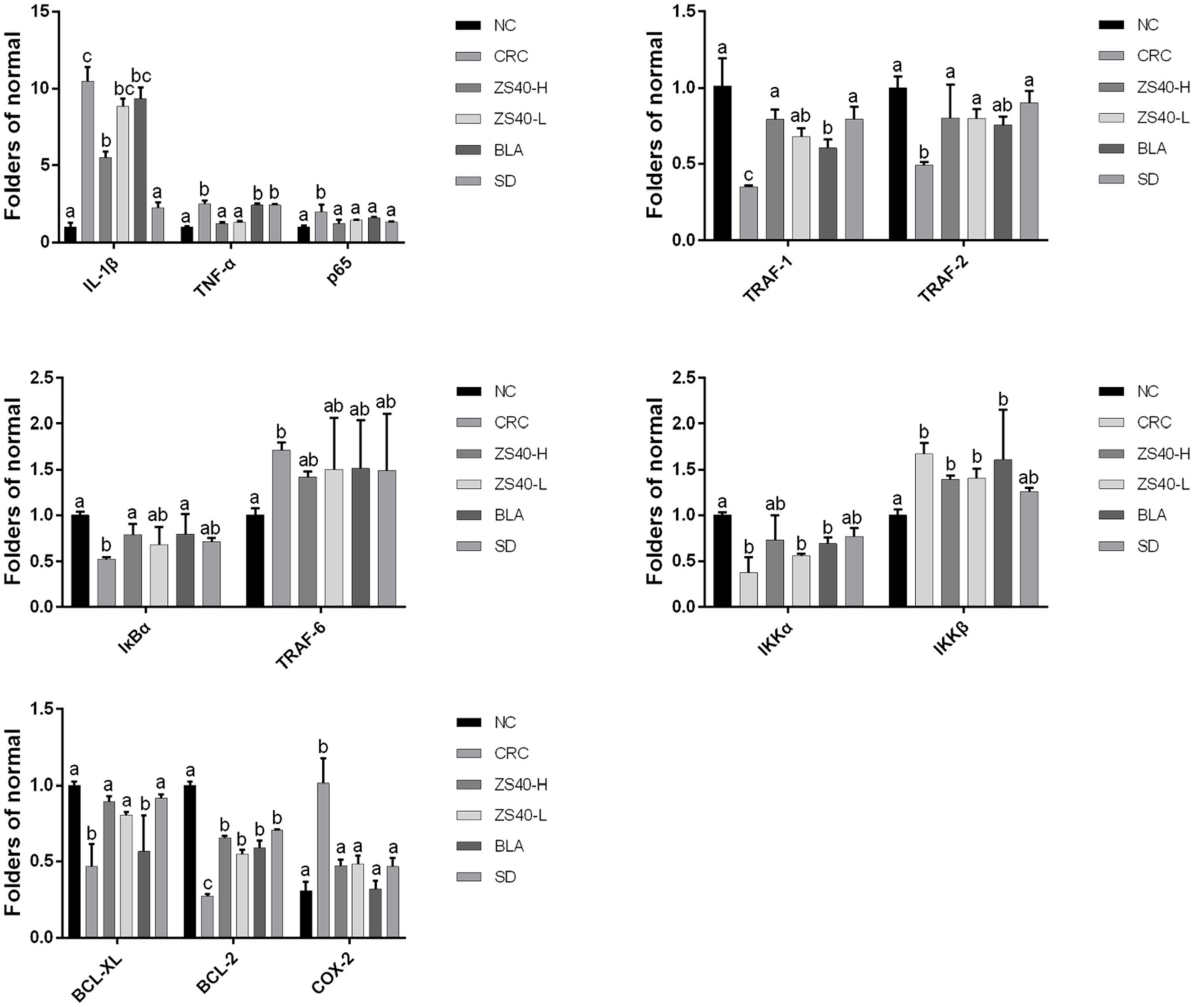
mRNA expression levels of inflammatory factors in colon tissue the difference in variance between the two groups was significant (*p* < 0.05), ^a–c^Mean values with different letters in the same column are significantly different (*p* < 0.05) according to Duncan’s honestly significantly different tests. NC, normal untreated mice; CRC, colon cancer model mice induced with AOM-DSS; ZS40-H, mice treated with high-dose *Lactobacillus fermentum* ZS40 (10^11^ CFU); ZS40-L, mice treated with low-dose *Lactobacillus fermentum* ZS40 (10^9^ CFU); BLA, mice treated with Bulgarian strain (10^11^ CFU); SD: mice treated with sulfasalazine (25%).

RT-qPCR analysis confirmed that the accumulation of inflammation caused an increase of IL-1β, TNF-α, p65, IKKβ, TRAF-6, and Cox-2 expression levels in the colon tissue of the CRC mice. At the same time, mRNA expression of TRAF-1/2, IκBα, IKKα, Bcl-2, and Bcl-xL decreased (Figure 6). An intervention with probiotic *Lactobacillus fermentum* ZS40 and anti-inflammatory drugs could reduce the expression levels of IL-1β, TNF-α, p65, IKKβ, TRAF-6, and Cox-2 in the colon tissue of CRC mice and increase those of TRAF-1/2, IκBα, IKKα, and Bcl-2. As for the mRNA expression level of Bcl-xL, SD had a better intervention effect, and high-dose *Lactobacillus fermentum* ZS40 showed better effects in relieving inflammation.

### Expression of key proteins in the NF-κB signaling pathway

**Figure 7:**
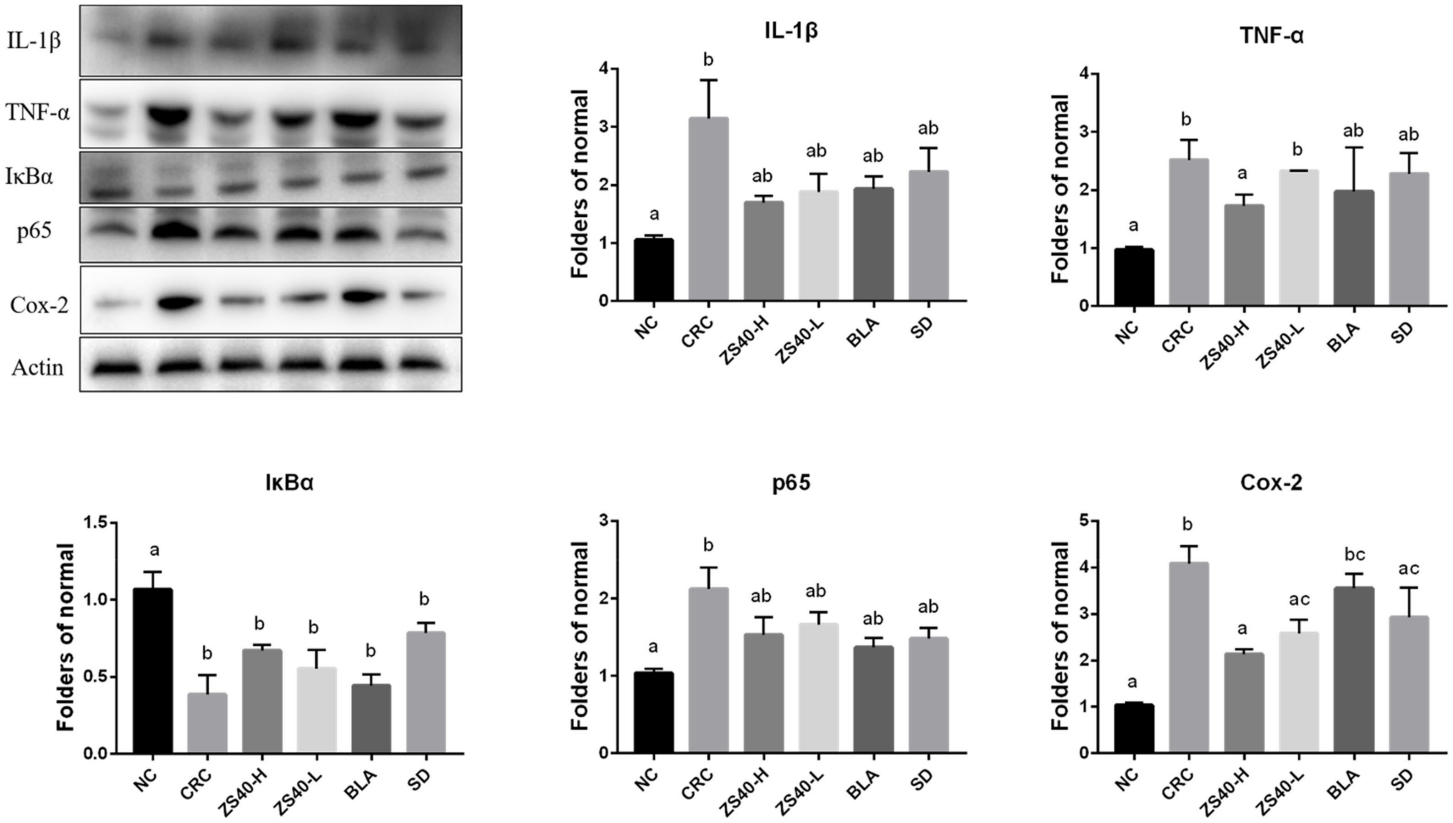
Expression of key proteins in the NFκB signaling pathway in colon tissue The difference in variance between the normal group and intervention was significant (*p* < 005), ^a–c^Mean values with different letters in the same column are significantly different (*p* < 005) according to Duncan’s honestly significantly different tests. NC, normal untreated mice; CRC, colon cancer model mice induced with AOM-DSS; ZS40-H, mice treated with high-dose *Lactobacillus fermentum* ZS40 (10^11^ CFU); ZS40-L, mice treated with low-dose *Lactobacillus fermentum* ZS40 (10^9^ CFU); BLA, mice treated with Bulgarian strain (10^11^ CFU); SD, mice treated with sulfasalazine (25%).

We analyzed the protein expression in mouse colon tissues using Western blot analysis (Figure 7). Compared with the NC group, the expression levels of IL-1β and TNF-α in colon tissue of the CRC group were increased. The ZS40-H, ZS40-L, BLA, and SD groups had reduced expression of inflammatory factors in colon tissues, among which the ZS40-H and SD groups showed the most obvious reduction effect. The expression levels of p65, IκBα, and Cox-2 in colon tissue also showed a decreasing trend in the ZS40-H, ZS40-L, BLA, and SD groups, as compared with the CRC group. However, the difference between the ZS40-H and SD groups was not obvious.

## Discussion

Epidemiological studies have confirmed that many tumors are caused by the repeated stimulation of inflammation (Li K. L. et al., 2019). Moreover, studies have shown that anti-inflammatory drugs can reshape the tumor immune environment and enhance the immune blocking effect (Pelly et al., 2021). Clinical studies have found that there may be a correlation between ulcerative colitis and colon cancer and that inflammatory cells are present in tumor biopsy samples (Wang et al., 2021). The clinical utility of inflammation-related biomarkers in routine blood tests has been reported in a variety of cancers, and Yamamoto, T. summarizes the prognostic impact of each inflammation-related marker on CRC (Yamamoto et al., 2021). Many previous studies have shown that long-term consumption of DSS solution can cause colitis, and the consumption of lactic acid bacteria has an intervening effect in preventing colon cancer caused by DSS (Wang C. Z. et al., 2018). The combined stimulation of AOM and DSS may induce colon cancer (Jeon et al., 2018). Research on the use of lactic acid bacteria to induce apoptosis in colon cancer cells is a popular research topic. In our study, the reason for successful interference of combined stimulation with AOM and DSS to induce colon cancer in mice can be attributed to the effect of long-term consumption of lactic acid bacteria.

Our results showed under the combined effect of AOM and DSS, obvious lump-like foreign bodies were found in the colon tissue of CRC mice, and the number and volume were larger. The intestinal tract is filled with a large amount of tissue mucus, the intestinal wall is thinned, and the blood vessels are obvious. It shows that the combined effect of AOM and DSS can form more obvious colon lesions in mice, and the intestinal inflammation is obvious. At the same time, the variation of lesions in the intestinal tissue of mice treated with *Lactobacillus fermentum* CQZS40 was improved. It is manifested as a smooth and elastic intestinal surface, and a decrease in the number and volume of foreign body masses in the intestinal tract. The results showed that ingestion of active *Lactobacillus fermentum* was effective in preventing colon cancer induced by the combination of AOM and DSS.

The active cytokine TNF-α is secreted by macrophages and lymphocytes activated by endotoxins. It has been reported that the main role of TNF-α in the body is not to kill tumors but rather to promote tumor development as an inflammatory factor (Lv et al., 2020). Moreover, TNF-α is highly correlated with colorectal cancer (Hu et al., 2019). TNF-α can induce tumor immunosuppressive microenvironment formation by activating inflammation-related signaling pathways, as well as promoting tumor angiogenesis and tumor spread (Shen et al., 2019). Interleukins transmit information; activate and regulate immune cells; mediate the activation, proliferation, and differentiation of T and B cells; and play an important role in inflammation (Lerner, 2020). Among them, IL-1β is an inflammatory cytokine, which is widely involved in various pathological damage processes such as human tissue destruction and edema formation. IL-8 is an important inflammatory mediator (Xiao et al., 2018). Moreover, when the body is affected by infection and certain autoimmune diseases, IL-8 significantly increases local inflammation and is elevated in serum. In the detection of cytokine levels, the mouse serum levels of TNF-α, IL-1β, and IL-8 were in line with expectations. The increase in the levels of these factors is the basic condition for promoting the occurrence of inflammation.

Together with the action of various factors that promote inflammation, the body triggers a series of acute reaction and fever reactions and can also activate endothelial cells to increase vascular permeability. MIP belongs to a class of chemokine (Chahar et al., 2018). MIP can activate granulocytes, regulate the adhesion of CD8+ T cells and vascular endothelial cells, participate in hematopoiesis regulation, and induce natural killer cell proliferation and activation. As an important factor that mediates the adhesion of leukocytes to vascular endothelial cells, vascular cell adhesion molecules play an important role in vascular injury. Studies have shown that the increase in MIP expression has an important role in the occurrence of vascular-related diseases (Yang et al., 2018). MIP-2 released during the early stage of inflammation chemotactically activates neutrophils to the site of inflammation, which then release a large number of proteolytic enzymes to cause inflammation (Subramanya et al., 2018). Vascular cell adhesion molecule (VCAM)-1 mediates cell-to-cell interactions and can induce the expression of VCAM-1 in the inflammatory tumor microenvironment by high expression of IL-1β (Shen et al., 2021). The results of the present research are similar to the abovementioned results of previous studies. The expression levels of MIP-1β and VCAM-1 were increased in the model group, but decreased after the *Lactobacillus fermentum* ZS40 intervention. This may be related to the reduction in inflammation factors TNF-α, IL-1β, and IL-8.

The expression level of protein indicators can express the condition of the body. The practical value of immunohistochemistry in tumor diagnosis and differential diagnosis has been generally recognized, and its accuracy rate can reach 50%–75% in the differential diagnosis of poorly differentiated or undifferentiated tumors. CD117 is a specific marker for gastrointestinal stromal tumors and is generally diagnosed in combination with CD34. At present, CD117 is mainly used in combination with CD34 in research of gastrointestinal stromal tumors. The adhesion of leukocytes and displacement of vascular endothelial cells to the inflammatory area are important processes in inflammation because of the interaction of adhesion molecules (Takayuki et al., 2019). Increasingly more research results show that CD34 molecules have an important role in mediating cell adhesion. In this process, CD34 can mediate the accumulation of leukocytes, initiate an inflammatory response, and simultaneously cooperate with chemokines to enhance the inflammatory response (Zhao D. W. et al., 2018). The CD117 protein is a type III tyrosine kinase growth factor, which results in strong membrane and cytoplasmic expressions in gastrointestinal inflammatory cells (Jelena et al., 2020). Our results support previous studies and provide evidence that high doses of *Lactobacillus fermentum* and sulfasalazine have better protection against colon cancer compared to *Lactobacillus bulgaricus* administration. This observation can be clearly observed in the significantly lower AOD levels, colon cancer morphology, and histological malignant changes in the probiotic-treated group compared with the AOM-DSS-treated group.

The NF-κB signaling pathway regulates key processes in the occurrence and development of various types of cancer (Zhu et al., 2018). The transcription factor NF-κB is a key mediator of inflammatory responses (Barnabei et al., 2021). NF-κB may be one of the most common regulators of cancer because of the extensive involvement of target genes and tissues. NF-κB protein has been detected in epithelial cells and macrophages of patients with ulcerative colitis, which provides evidence for constitutive NF-κB activation (Tong et al., 2019). Under the stimulation of pro-inflammatory cytokines TNF-α, interleukin IL-8, and other extracellular factors, IKKβ is activated after phosphorylation of TAK1 protein, resulting in the degradation of the IκB protein and the release of NF-κB dimers. Pro-inflammatory cytokines also induce p65 and activate the NF-κB pathway (Yang et al., 2019). Our detection of serum inflammatory cytokine levels and the detection of intestinal tissue gene and protein expression levels are in line with previous studies, and provide evidence that under the combined induction of AOM-DSS, the NF-κB signaling pathway in mice is activated, which is manifested in the body increased levels of inflammation. Compared with the CRC group, the *Lactobacillus fermentum*-treated group had lower detection levels of inflammatory signaling pathway stimulators IL-1β and TNF-α, and increased expression of the activating protein IKKβ, resulting in the degradation of IκBα and the increase in the expression of aggregate NF-κB. The above results can indicate that the role of *Lactobacillus fermentum* can protect mice from reducing inflammatory stimulation.

At the same time, studies have found that when the NF-kB signaling pathway is activated, a series of changes in the expression of oncogenes and proteins will occur. The expression of Cox-2 is closely related to NF-kB. It has been reported that in the research on Cox-2 selective inhibitors, it has a clear efficacy similar to NSAIDs in terms of analgesia and anti-inflammatory, and can protect gastrointestinal cells and prevent ulcer formation (El-Malah et al., 2022). Kitagawa et al. compared the expression of Cox-2 in colon cancer and normal colon tissue, and the results showed that Cox-2 was overexpressed in colon cancer tissue, while the expression of Cox-2 in normal colon mucosa was negative (Kitagawa et al., 2022). Bcl-2 is a major inhibitor of apoptosis gene. Theoretically, when it shows a high-intensity level, it causes a stronger result of inhibiting apoptosis. Corresponding results showed that the greater the probability of tumor cells evading apoptosis, the higher the malignancy of the tumor. The expression of Bcl-2 in various tumors has been confirmed. For example, Abdel-Wahab et al. showed that Bcl-2 protein expression is positively correlated with the differentiation of colorectal cancer and is a useful indicator for judging the malignancy of colorectal cancer (Abdel-Wahab et al., 2021). Bcl-xL is an anti-apoptotic protein belonging to the Bcl-2 family, which is involved in the regulation of cell apoptosis. Play an important role, a large number of studies have reported that Bcl-xL is highly expressed in colorectal cancer (Ramesh et al., 2021). The changes of Cox-2, Bcl-2, Bcl-XL are also reflected in our detection results, Cox-2, Bcl-2. The higher level of expression of Bcl-XL was found after the activation of the signaling pathway. This trend was suppressed in the *Lactobacillus fermentum* treatment group. This result is also in line with the experimental expectation.

Activation of the NF-κB canonical pathway ultimately leads to increased expression of NF-κB target genes such as IL-8, Bcl-2, and Cox-2. High expression of these pro-inflammatory factors, in turn, further exacerbates the stimulatory pathways that continue to affect cell proliferation or renewal. Therefore, NF-κB may contribute to the occurrence of colitis-associated colon cancer by maintaining a continuous inflammatory process in the intestinal mucosa. The direct and indirect effects of this transcription factor complex on tumorigenesis and progression have been validated in various animal models such as hepatocellular carcinoma, gastric cancer, and lung cancer (Ji et al., 2018; Wang J. J. et al., 2018, 2020). In our study, the *Lactobacillus fermentum* treatment group blocked the continuous development of the signaling pathway by affecting the important turning points of the NF-κB classical pathway, such as IκBα, p65, and IKKβ, as well as the release of pro-inflammatory factors.

## Conclusion

Our present study showed that the consumption of *Lactobacillus fermentum* CQZS40 can reduce the occurrence of colon cancer tumors induced by AOM and DSS by inhibiting the NF-κB classical signaling pathway. The present results confirmed that experimental animals in the ZS40-H intervention group showed a decrease in the number of intestinal tissue cysts and a decrease in the level of intestinal inflammation compared with the CRC group, by affecting the release of inflammatory cytokines, gene and protein expression in colon tissue. Moreover, both high and low doses of Lactobacillus fermentum ZS40 could effectively inhibit the NF-κB signaling pathway. Among them, high doses of *Lactobacillus fermentum* ZS40 were more effective in inhibiting pro-inflammatory factors and regulating key proteins in the signaling pathway. These results may be related to the regulation of intestinal flora in the body after the ingestion of *Lactobacillus fermentum* ZS40. The present research finding is of great value for subsequent research on related areas and has inspired the authors’ interest in researching the mechanism of *Lactobacillus fermentum* ZS40 in the intestine.

## Data availability statement

The original contributions presented in the study are included in the article/Supplementary material, further inquiries can be directed to the corresponding author.

## Ethics statement

This study was approved by the Ethics Committee of Chongqing Collaborative Innovation Center for Functional Food (20190902B, Chongqing, China) and followed the national standard of the People’s Republic of China (GB/T 35892-2018) laboratory animal guidelines for ethical review of animal welfare.

## Author contributions

JL and SW performed the majority of the experiments and wrote the manuscript. RY and XL contributed to the data analysis. XZ designed and supervised the study, and checked the final manuscript. All authors contributed to the article and approved the submitted version.

## Funding

This research was funded by the Chongqing University Innovation Research Group Project (CXQTP20033) and the Science and Technology Project of Chongqing Education Commission (KJQN202001604).

## Conflict of interest

The authors declare that the research was conducted in the absence of any commercial or financial relationships that could be construed as a potential conflict of interest.

## Publisher’s note

All claims expressed in this article are solely those of the authors and do not necessarily represent those of their affiliated organizations, or those of the publisher, the editors and the reviewers. Any product that may be evaluated in this article, or claim that may be made by its manufacturer, is not guaranteed or endorsed by the publisher.

## Abbreviations

AOM-DSS: azoxymethane-dextran sodium sulfate
MRS: Man Rogosa
IL-1β: interleukin 1β
IL-8: interleukin 8
TNF-α: tumor necrosis factor-α
MIP-1β: macrophage inflammatory protein 1β
VCAM-1: vascular endothelial cell adhesion molecule 1
TRAF: TNF receptor-associated factor
IκBα: nuclear factor inhibitor protein
IKKα/β: IκB kinase
Bcl-XL/2: B-cell lymphoma/Leukemia
Cox-2: Cyclooxygenase-2
