# Supplementary figures and images for: Effect of Lactobacillus fermentum ZS40 on the NF-κB signaling pathway in an azomethane-dextran sulfate sodium-induced colon cancer mouse model
Source: Front Microbiol. 2022 Sep 26;13:953905. doi: 10.3389/fmicb.2022.953905 (PMC9549056; doi:10.3389/fmicb.2022.953905)

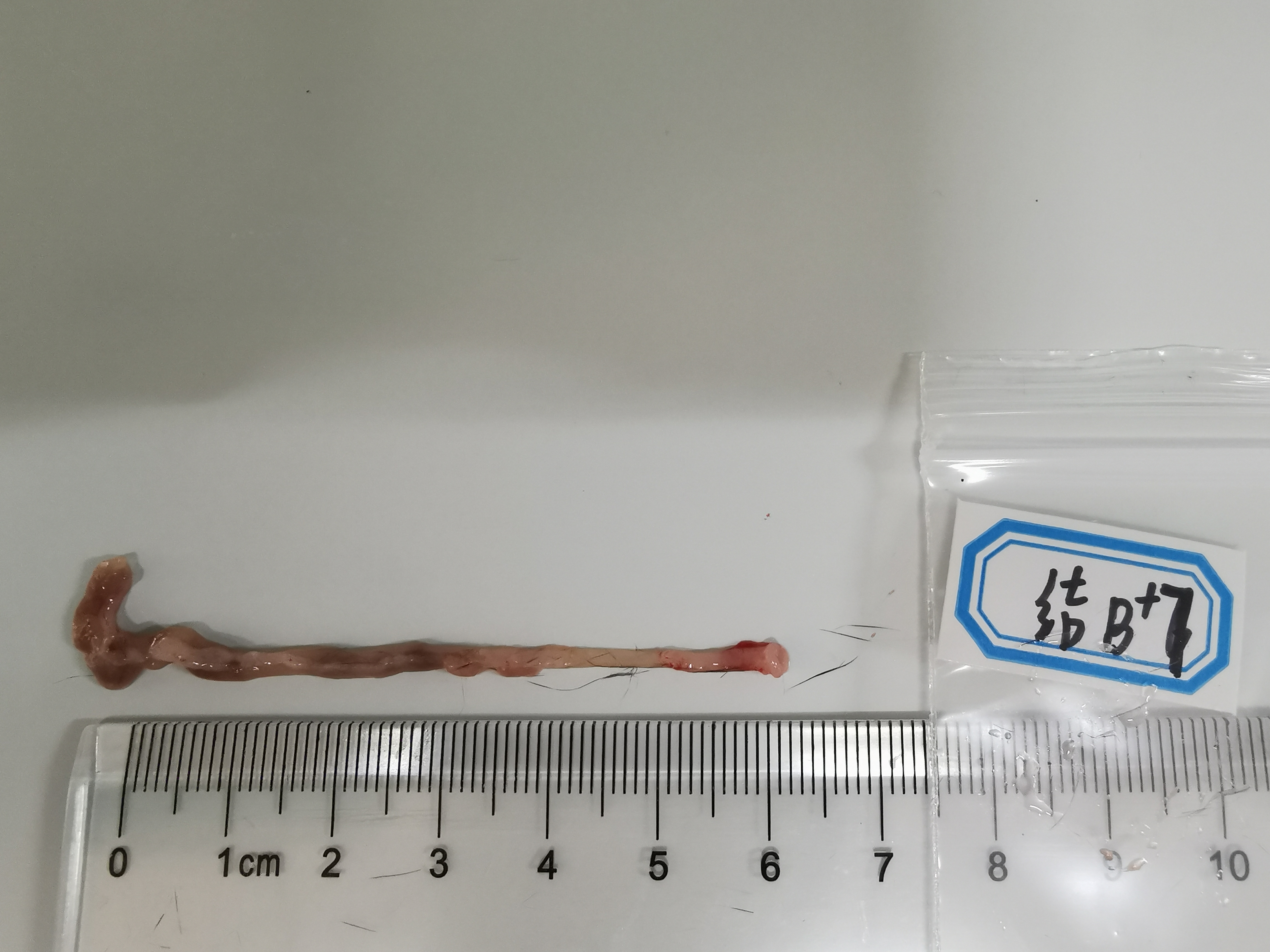

Supplement: Supplementary file 1 [file Data_Sheet_1.ZIP › Raw data/Raw data/Raw data/Figure 3. Colon picture and Colon weight/Picture/BLA.jpg]

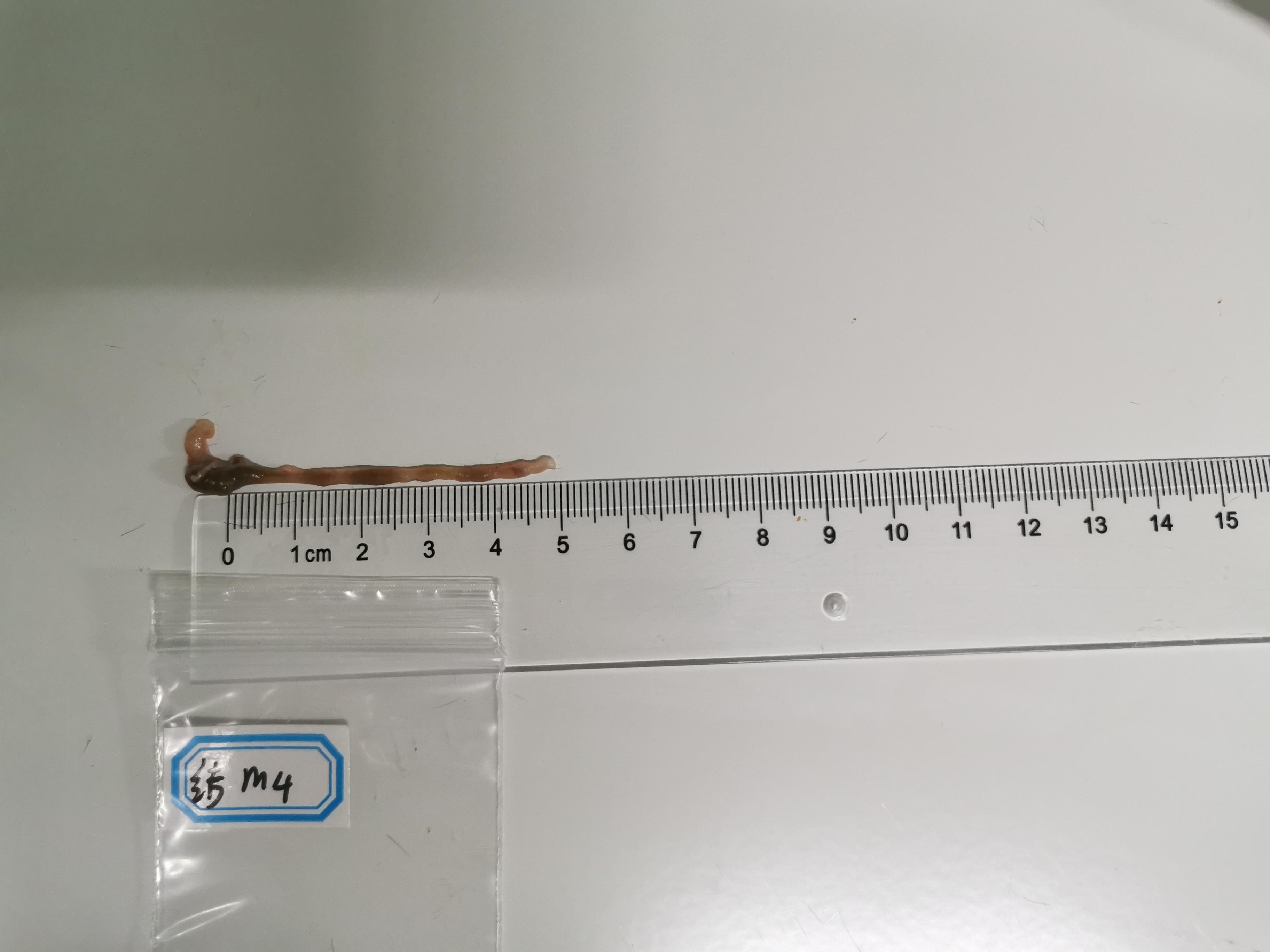

Supplement: Supplementary file 1 [file Data_Sheet_1.ZIP › Raw data/Raw data/Raw data/Figure 3. Colon picture and Colon weight/Picture/M.jpg]

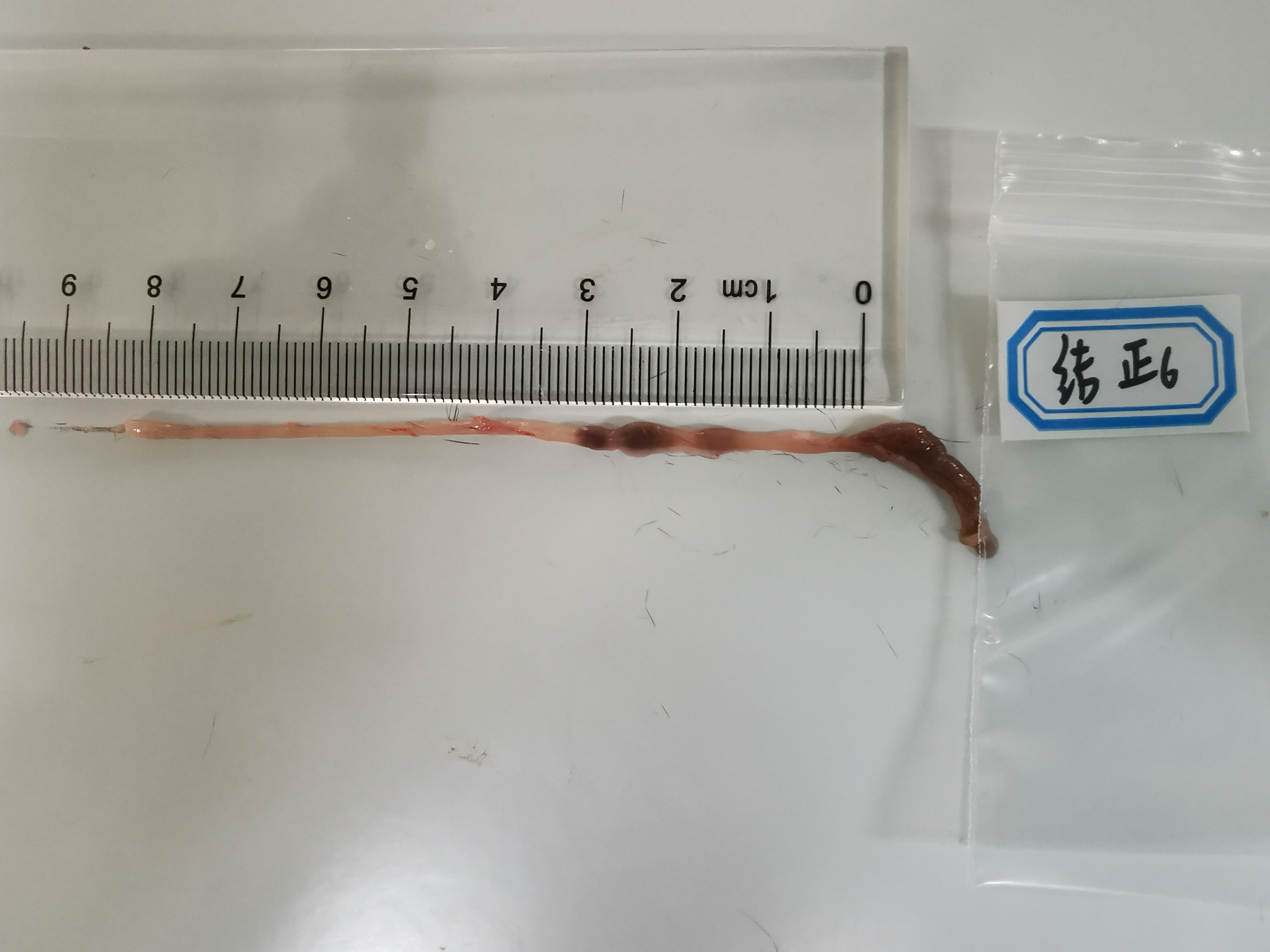

Supplement: Supplementary file 1 [file Data_Sheet_1.ZIP › Raw data/Raw data/Raw data/Figure 3. Colon picture and Colon weight/Picture/NC.jpg]

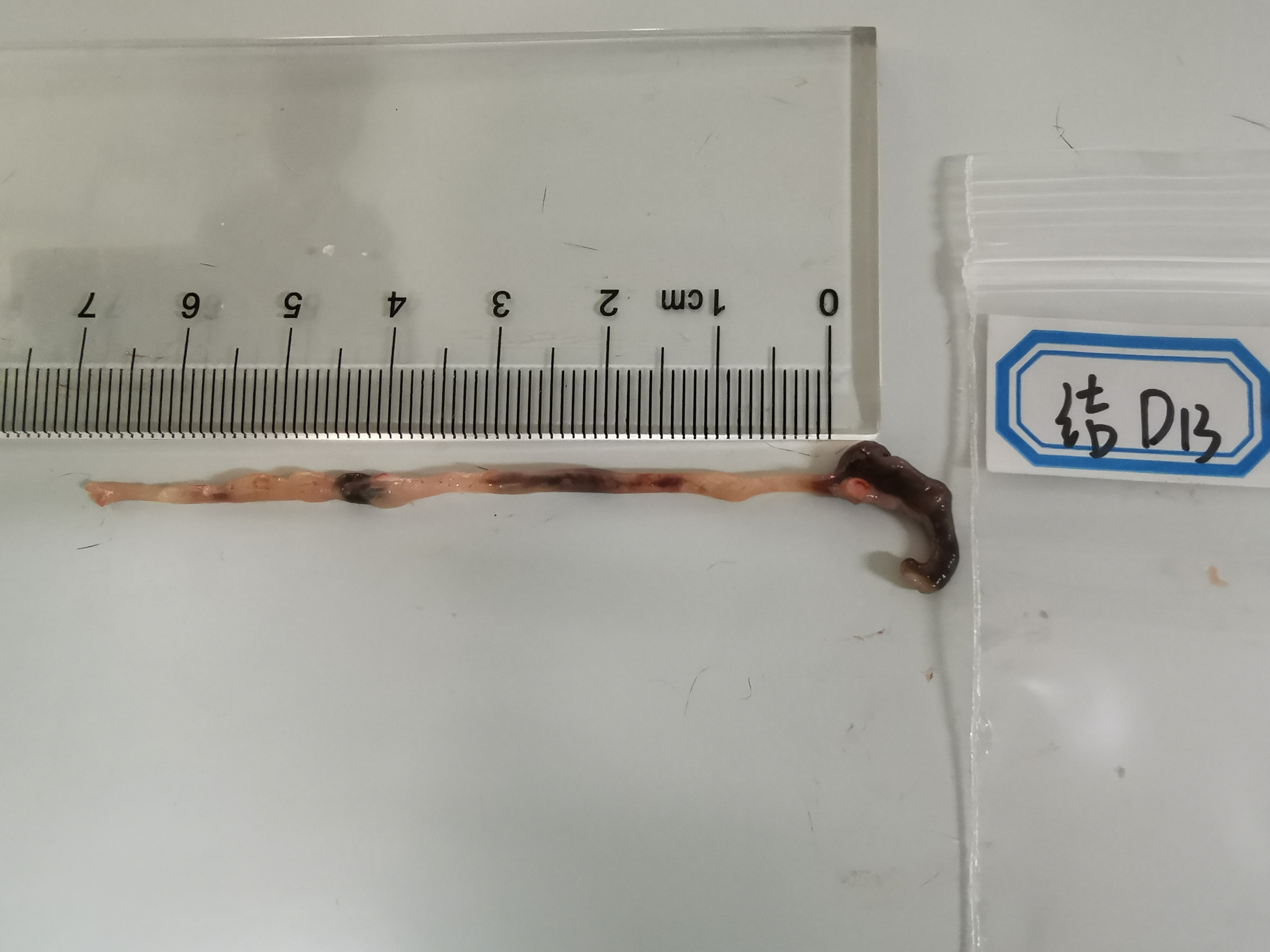

Supplement: Supplementary file 1 [file Data_Sheet_1.ZIP › Raw data/Raw data/Raw data/Figure 3. Colon picture and Colon weight/Picture/SD.jpg]

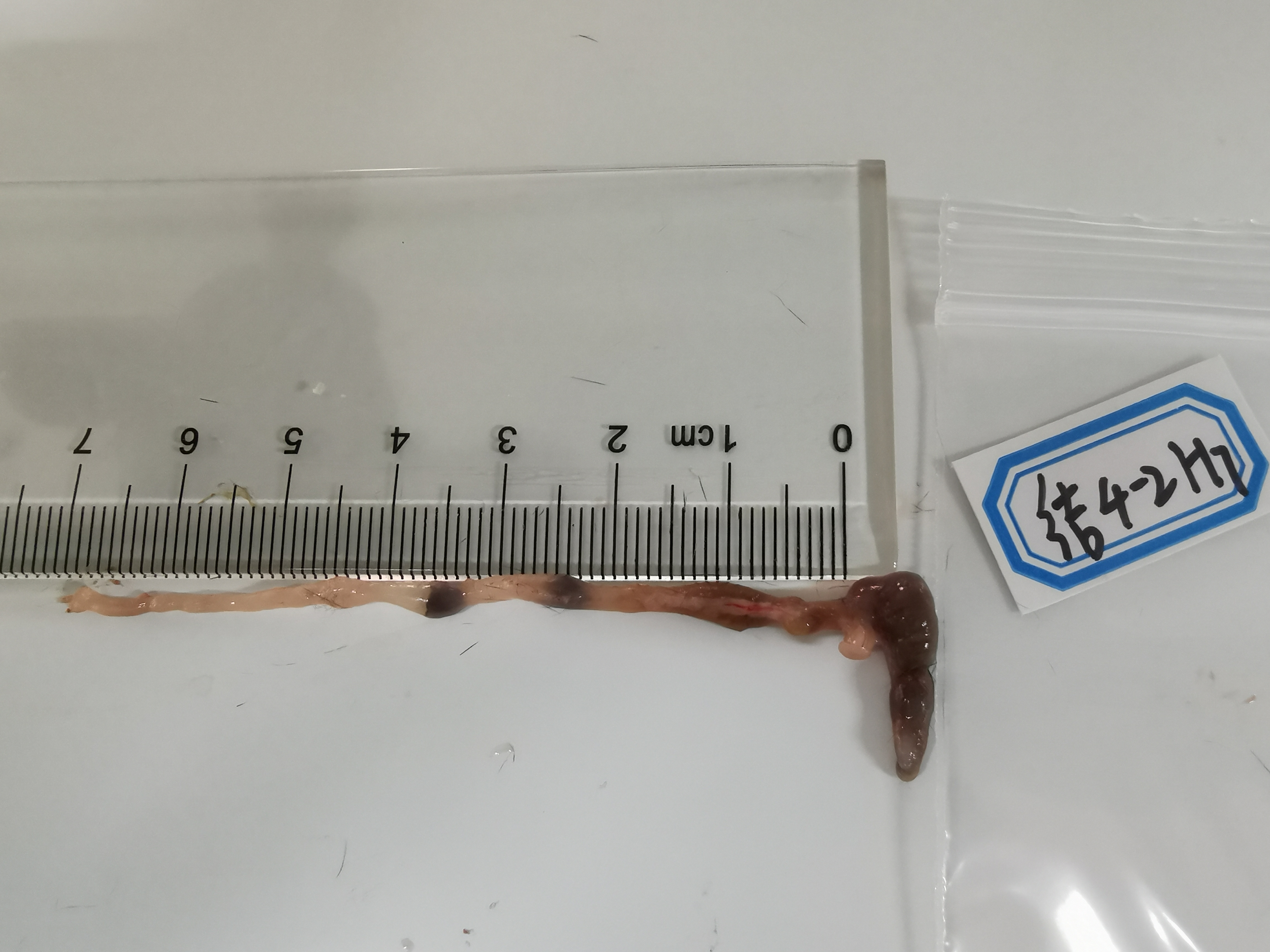

Supplement: Supplementary file 1 [file Data_Sheet_1.ZIP › Raw data/Raw data/Raw data/Figure 3. Colon picture and Colon weight/Picture/ZS40-H.jpg]

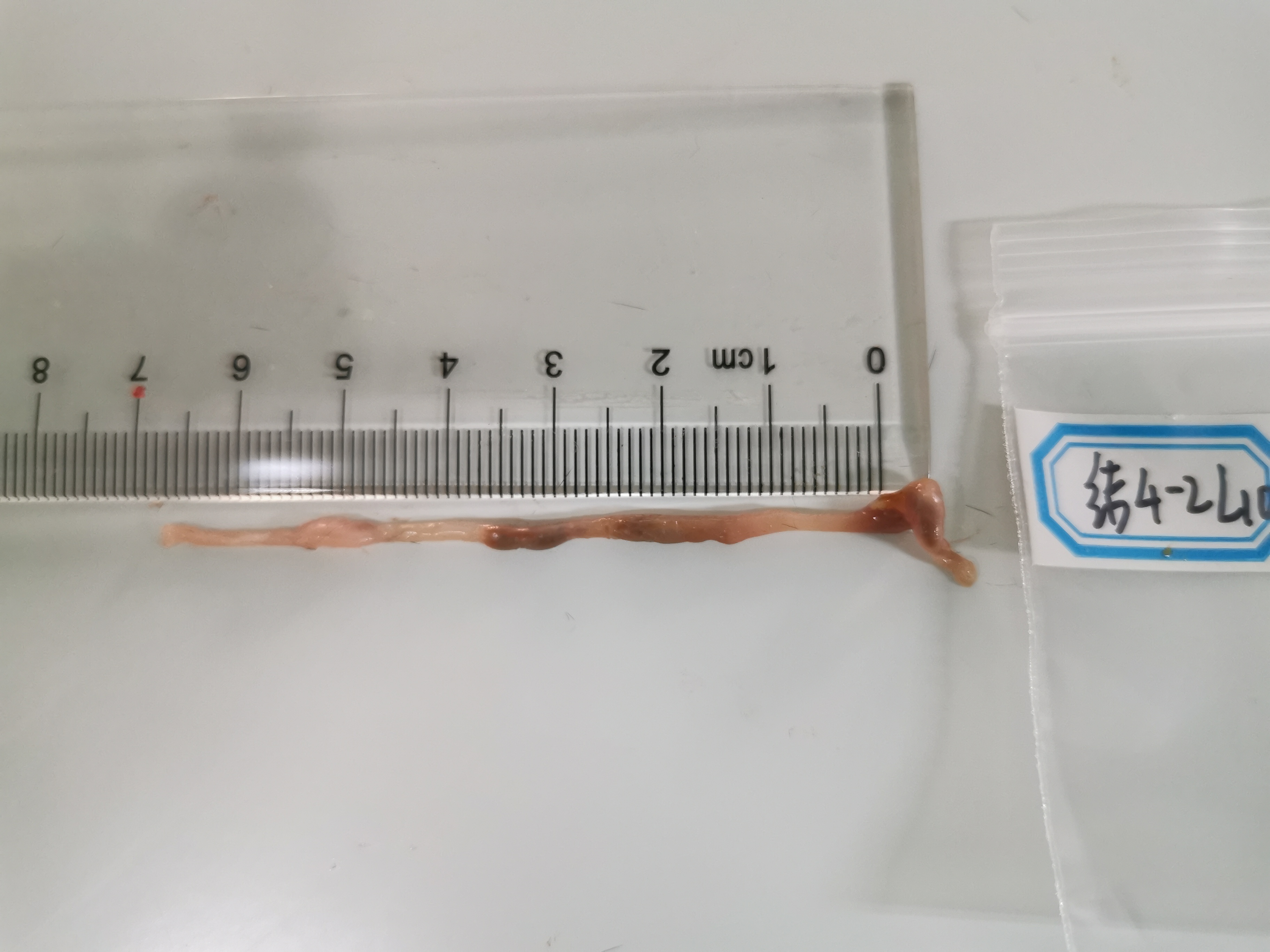

Supplement: Supplementary file 1 [file Data_Sheet_1.ZIP › Raw data/Raw data/Raw data/Figure 3. Colon picture and Colon weight/Picture/ZS40-L.jpg]

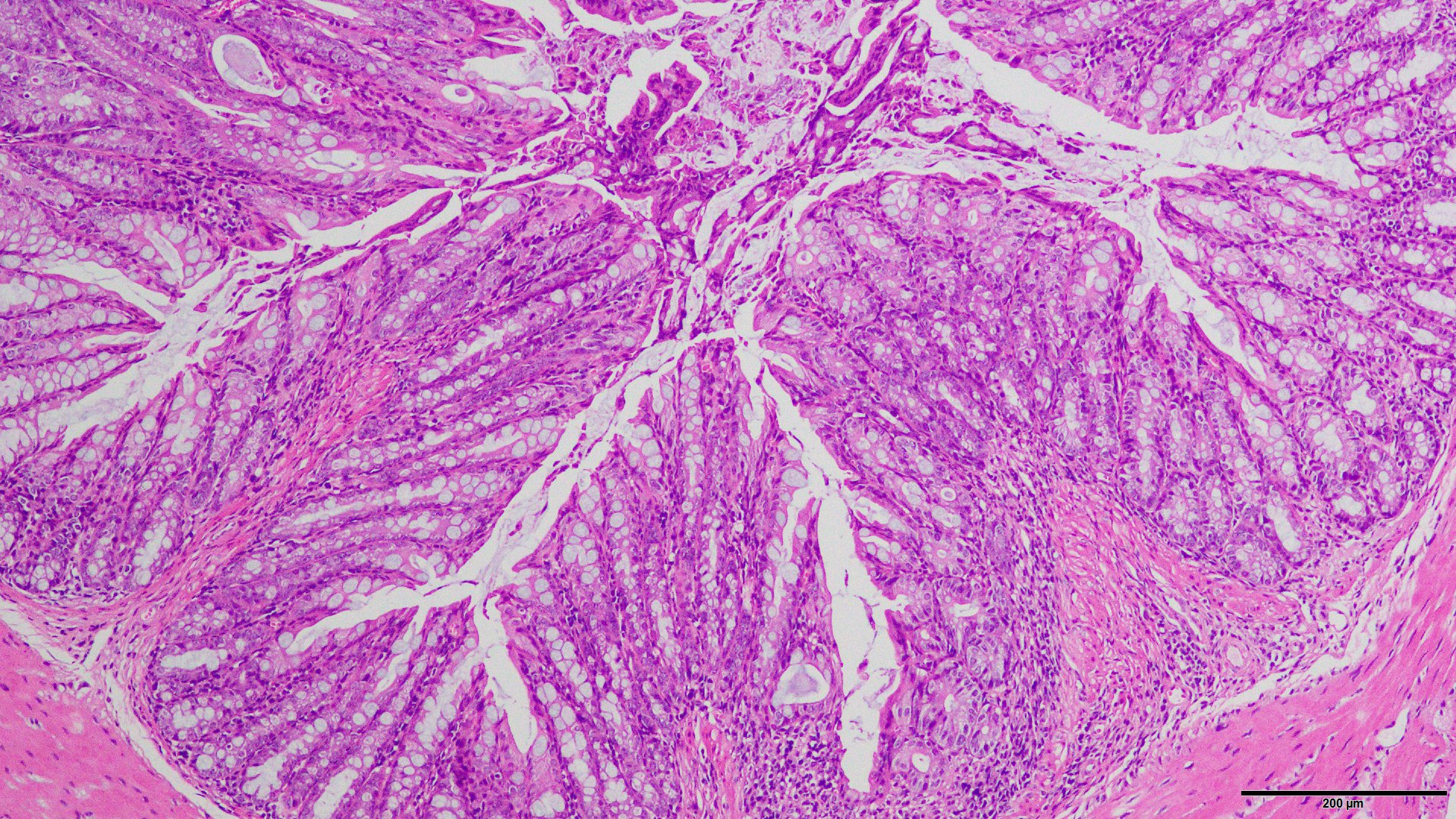

Supplement: Supplementary file 1 [file Data_Sheet_1.ZIP › Raw data/Raw data/Raw data/Figure 4. H&E/BLA.jpg]

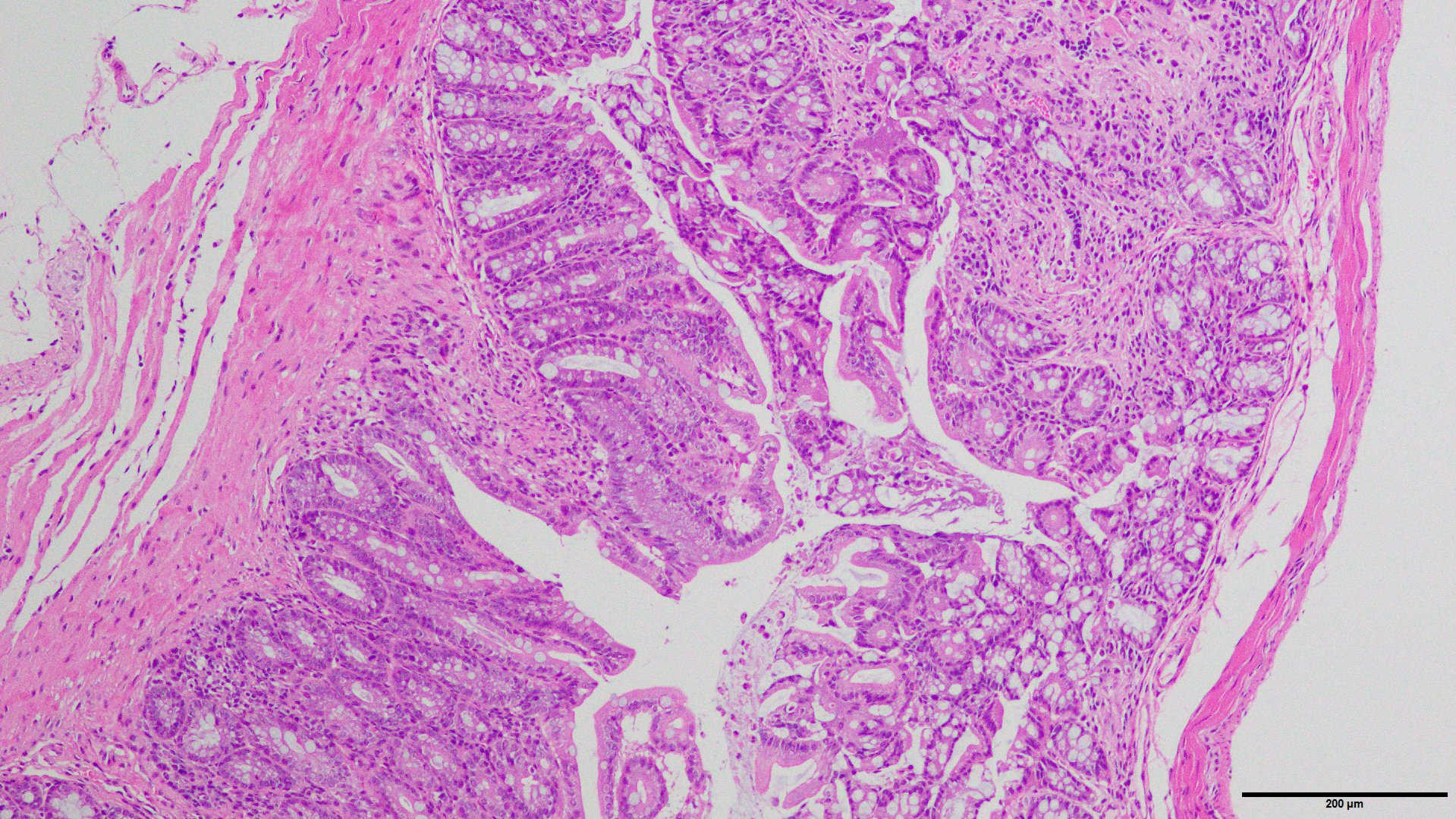

Supplement: Supplementary file 1 [file Data_Sheet_1.ZIP › Raw data/Raw data/Raw data/Figure 4. H&E/CRC.jpg]

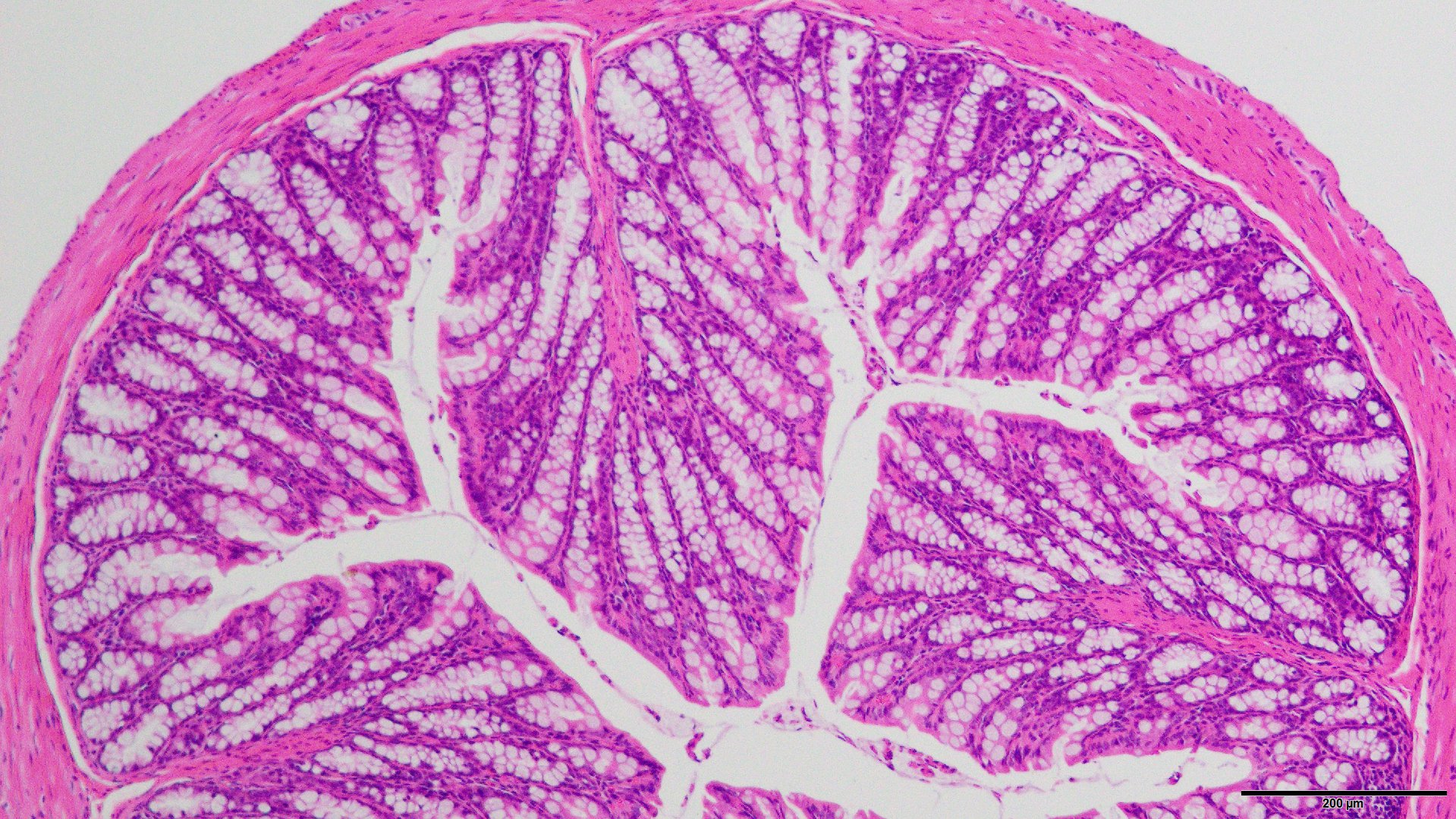

Supplement: Supplementary file 1 [file Data_Sheet_1.ZIP › Raw data/Raw data/Raw data/Figure 4. H&E/NC.jpg]

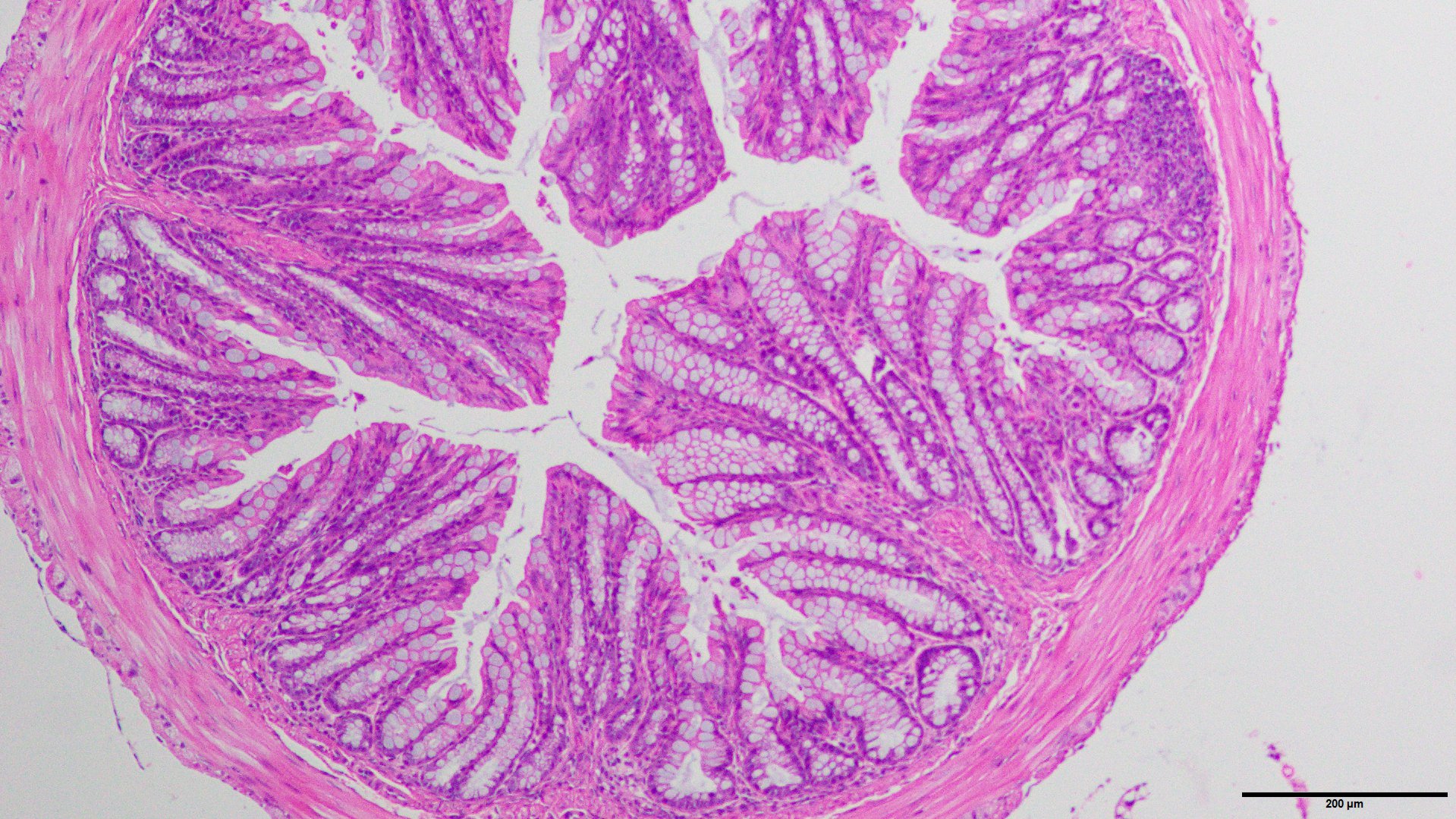

Supplement: Supplementary file 1 [file Data_Sheet_1.ZIP › Raw data/Raw data/Raw data/Figure 4. H&E/SD.jpg]

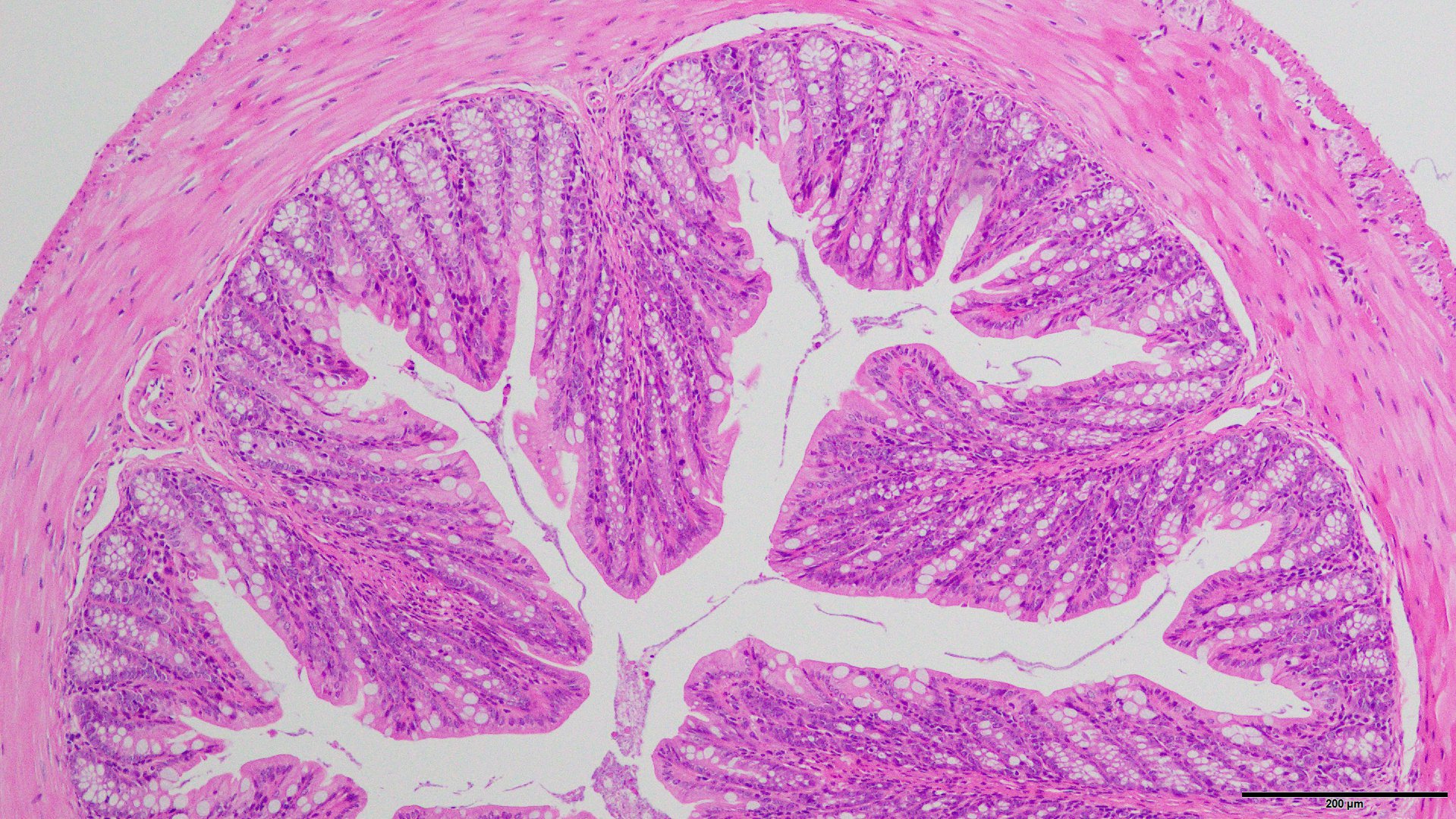

Supplement: Supplementary file 1 [file Data_Sheet_1.ZIP › Raw data/Raw data/Raw data/Figure 4. H&E/ZS40-H.jpg]

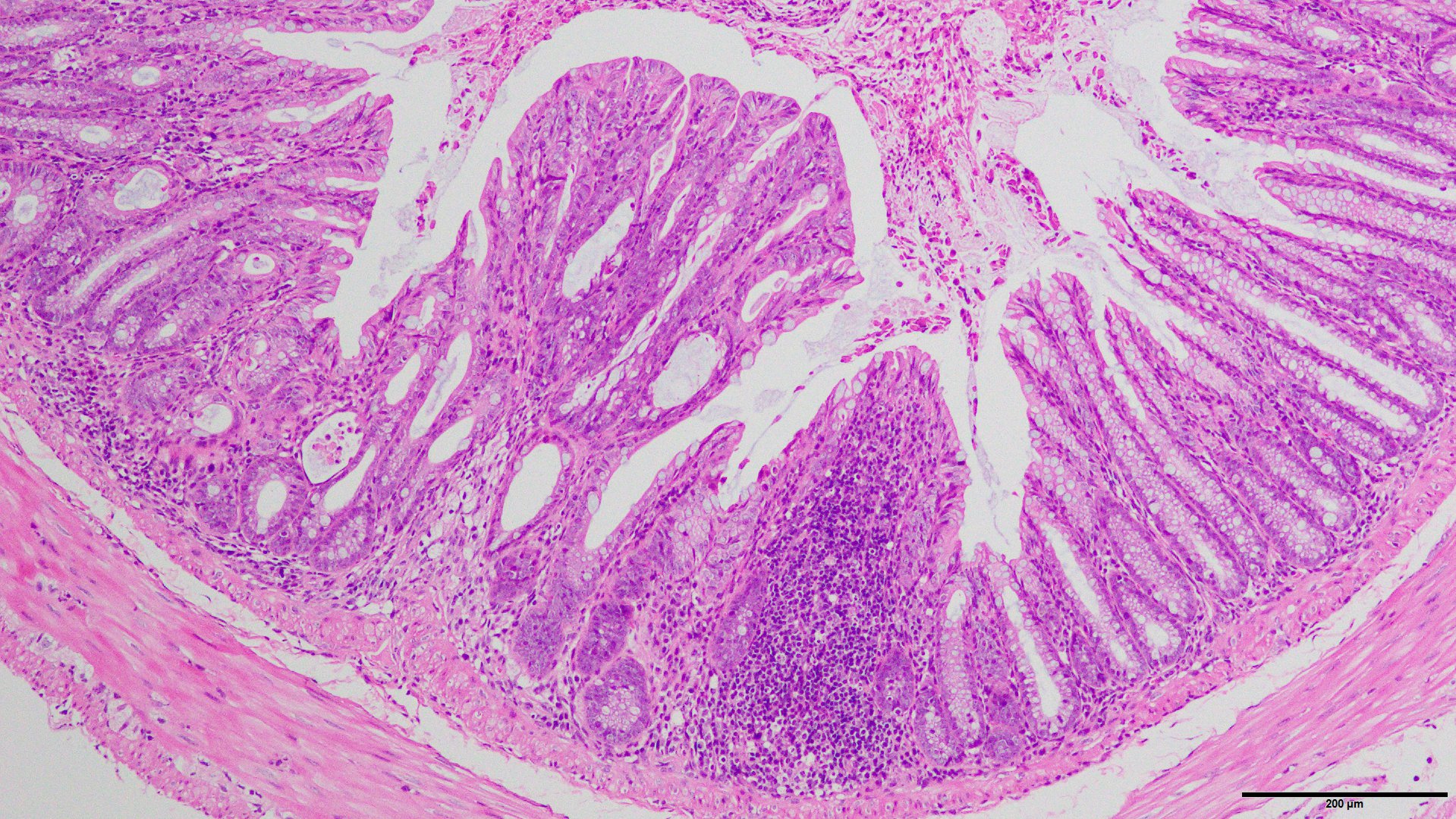

Supplement: Supplementary file 1 [file Data_Sheet_1.ZIP › Raw data/Raw data/Raw data/Figure 4. H&E/ZS40-L.jpg]

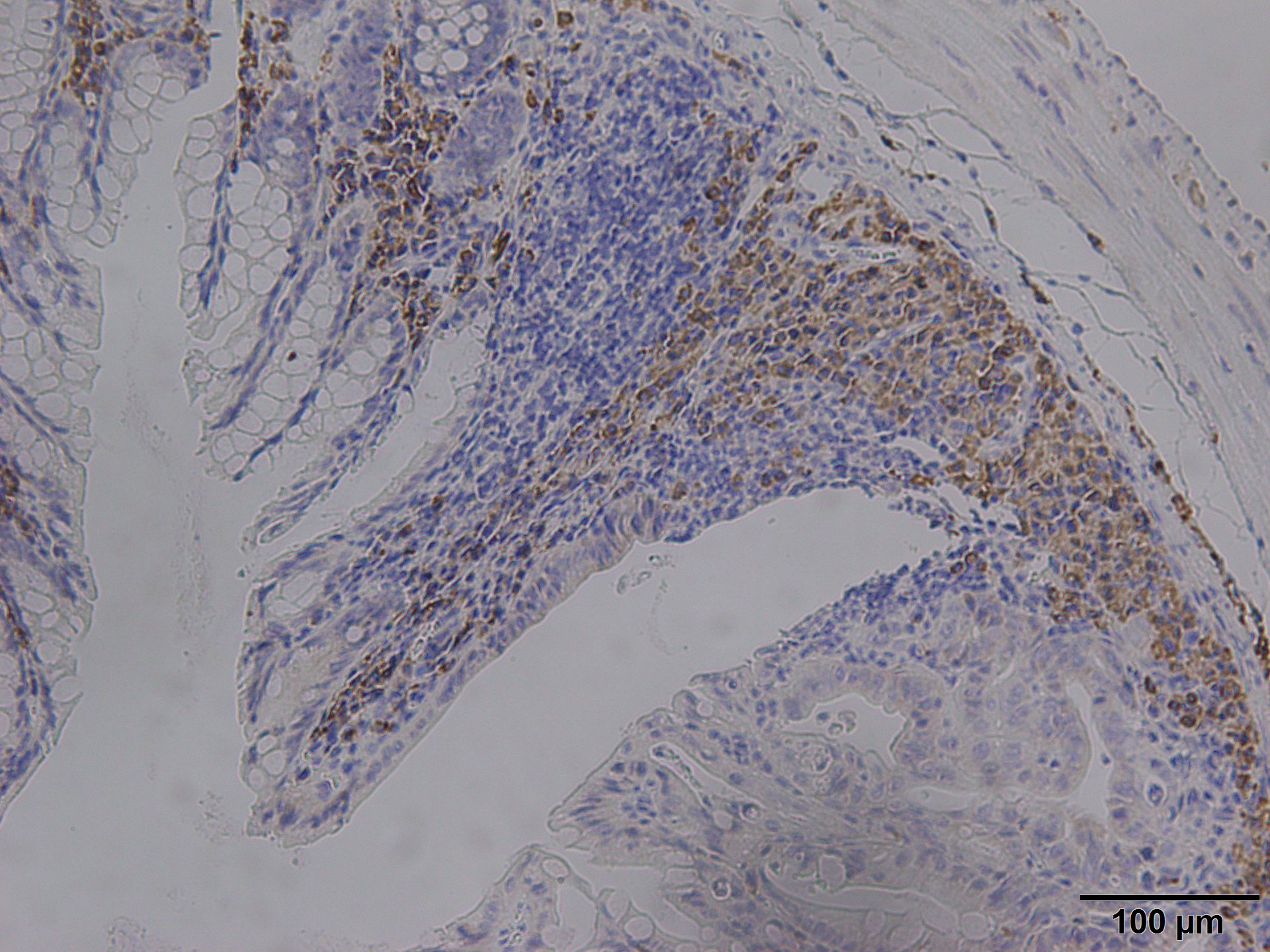

Supplement: Supplementary file 1 [file Data_Sheet_1.ZIP › Raw data/Raw data/Raw data/Figure 6. immunohistochemical staining/CD117/BLA.jpg]

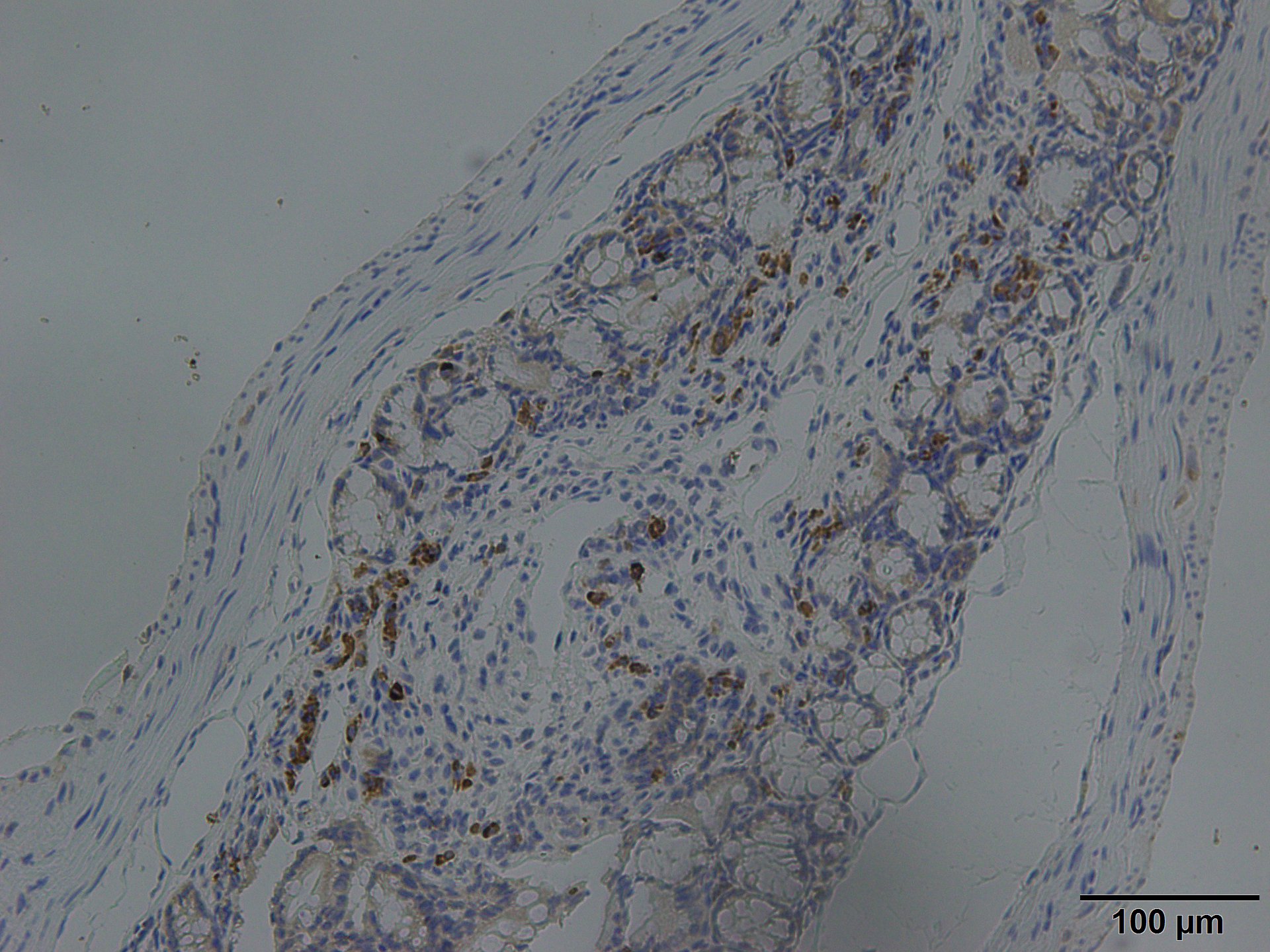

Supplement: Supplementary file 1 [file Data_Sheet_1.ZIP › Raw data/Raw data/Raw data/Figure 6. immunohistochemical staining/CD117/CRC.jpg]

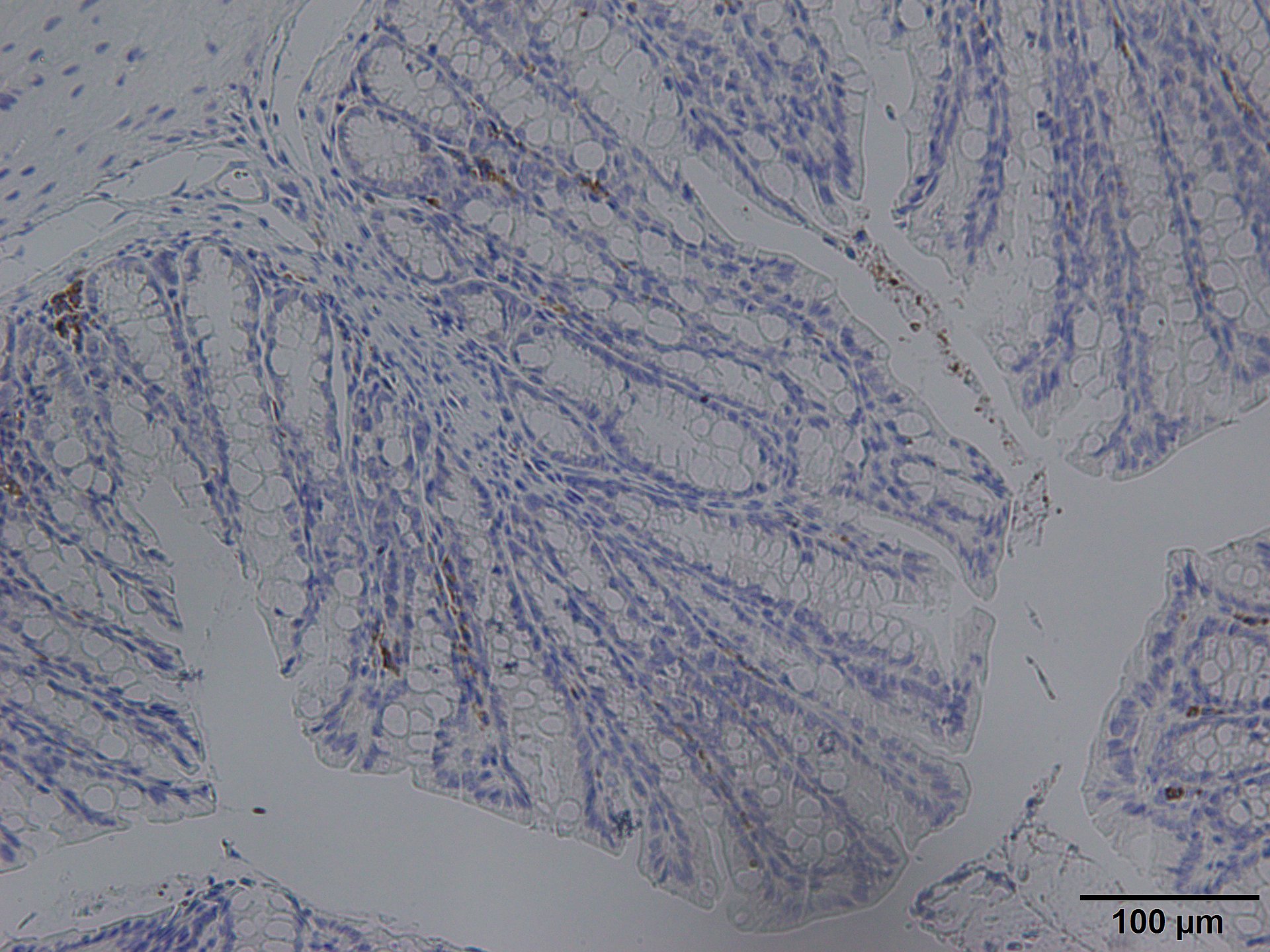

Supplement: Supplementary file 1 [file Data_Sheet_1.ZIP › Raw data/Raw data/Raw data/Figure 6. immunohistochemical staining/CD117/NC.jpg]

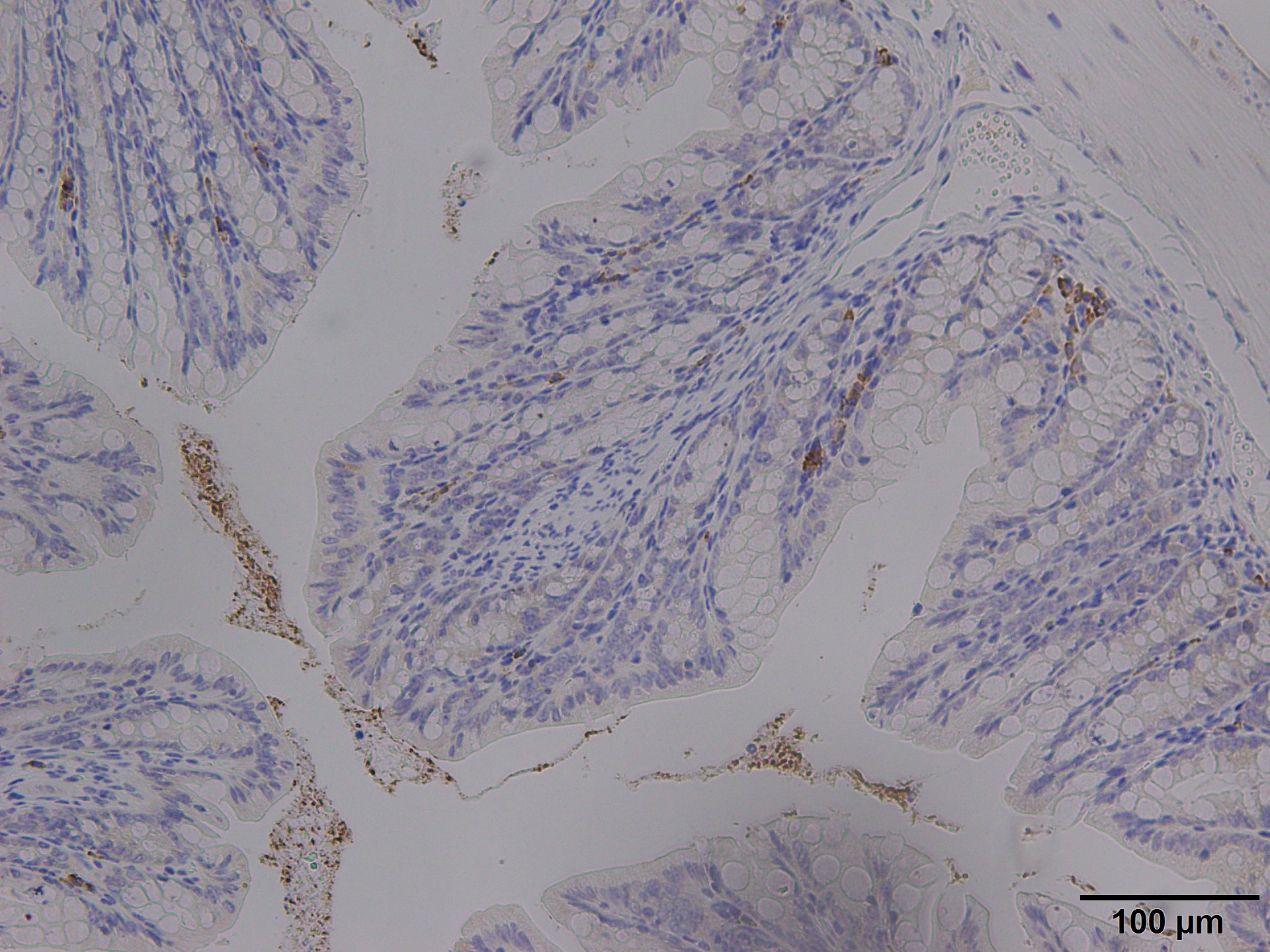

Supplement: Supplementary file 1 [file Data_Sheet_1.ZIP › Raw data/Raw data/Raw data/Figure 6. immunohistochemical staining/CD117/SD.jpg]

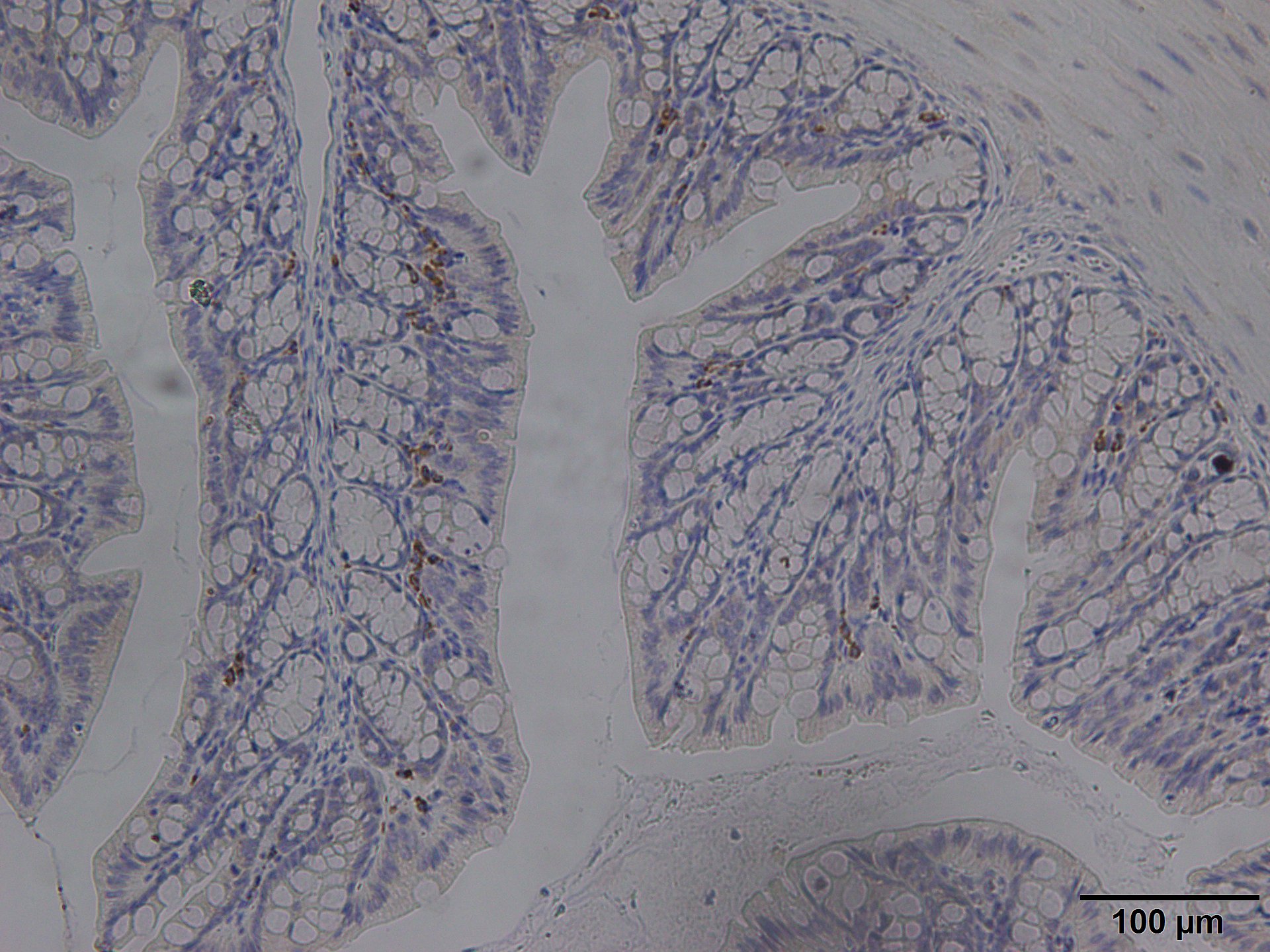

Supplement: Supplementary file 1 [file Data_Sheet_1.ZIP › Raw data/Raw data/Raw data/Figure 6. immunohistochemical staining/CD117/ZS40-H.jpg]

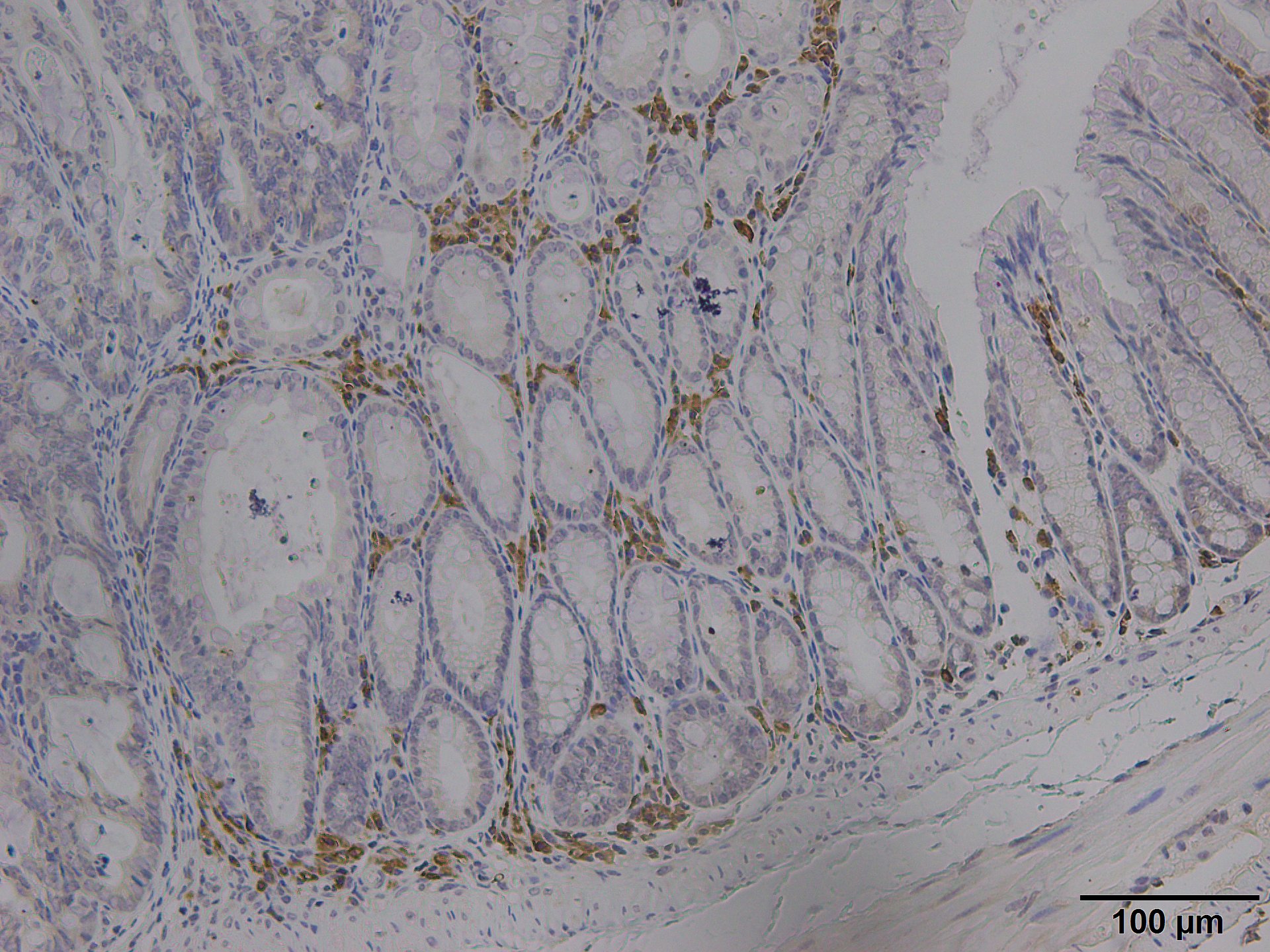

Supplement: Supplementary file 1 [file Data_Sheet_1.ZIP › Raw data/Raw data/Raw data/Figure 6. immunohistochemical staining/CD117/ZS40-L.jpg]

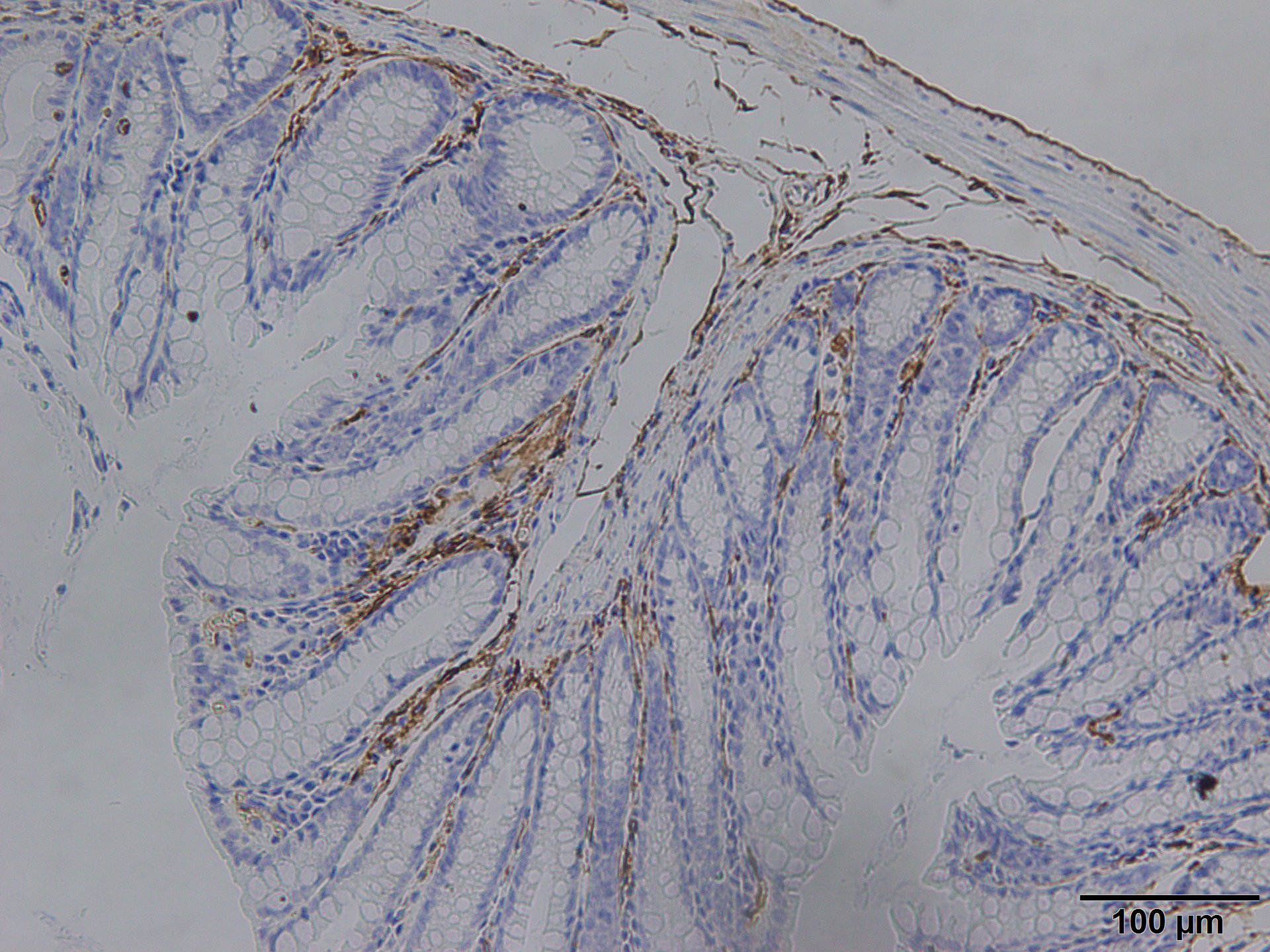

Supplement: Supplementary file 1 [file Data_Sheet_1.ZIP › Raw data/Raw data/Raw data/Figure 6. immunohistochemical staining/CD34/BLA.jpg]

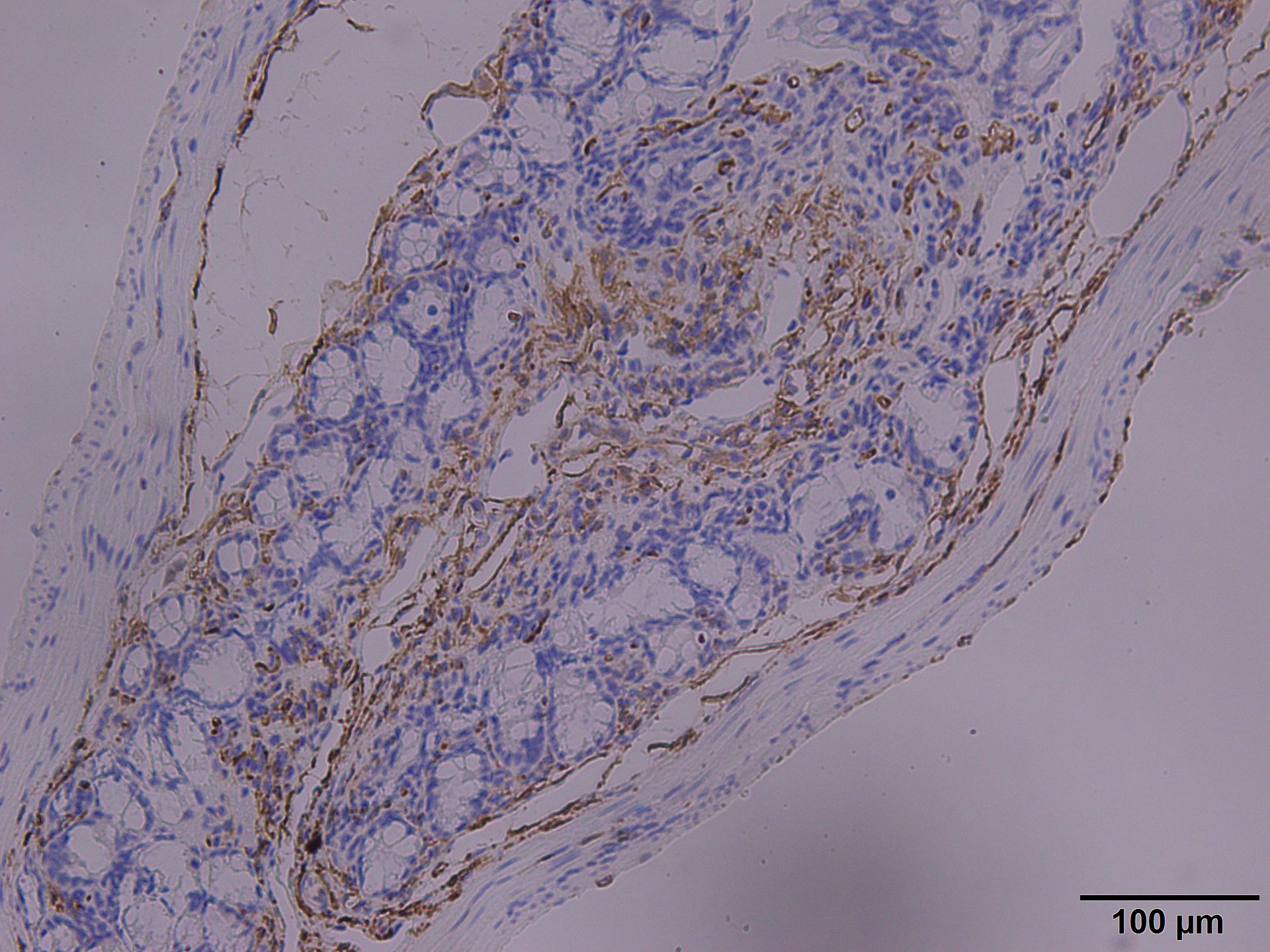

Supplement: Supplementary file 1 [file Data_Sheet_1.ZIP › Raw data/Raw data/Raw data/Figure 6. immunohistochemical staining/CD34/CRC.jpg]

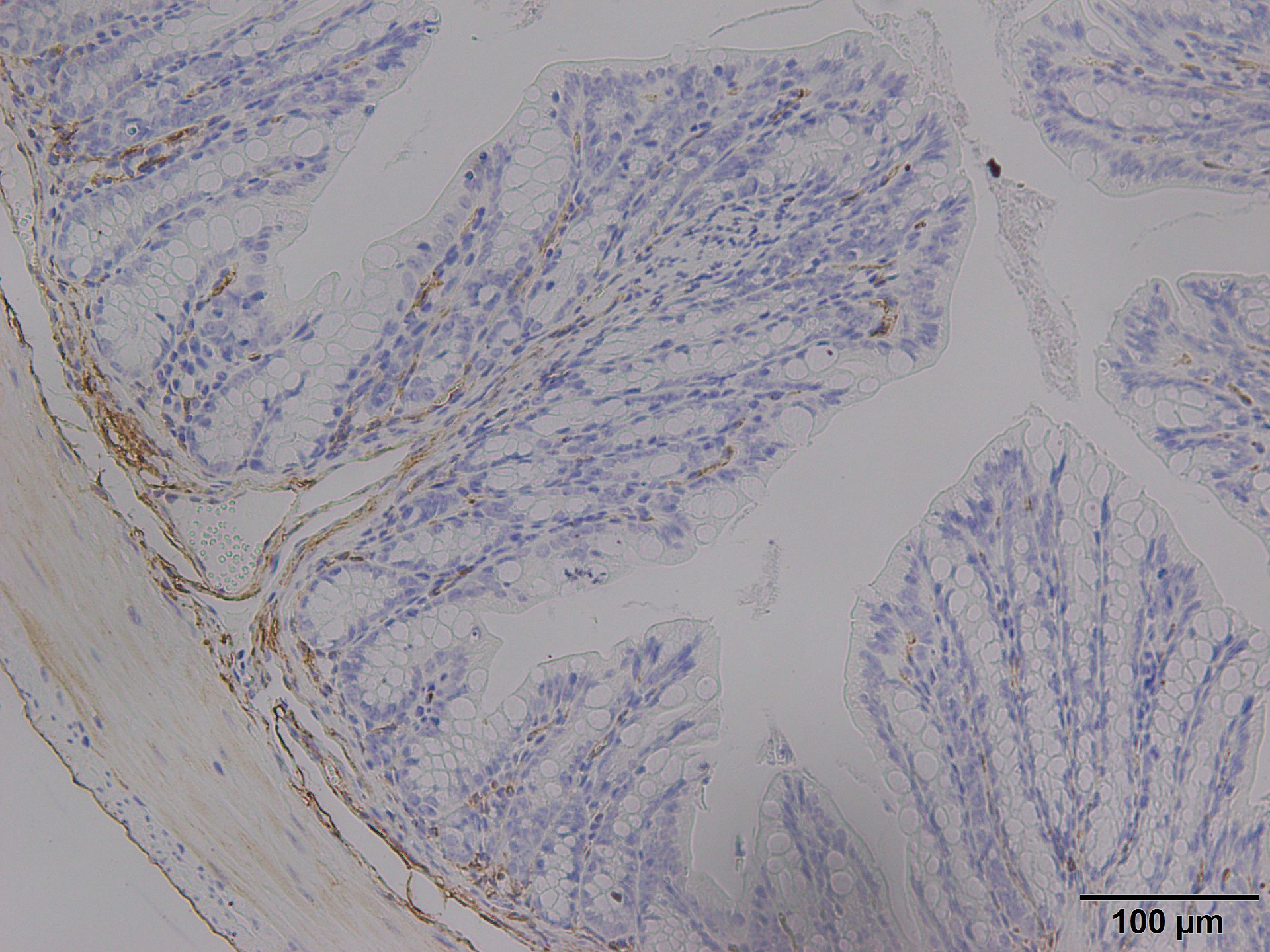

Supplement: Supplementary file 1 [file Data_Sheet_1.ZIP › Raw data/Raw data/Raw data/Figure 6. immunohistochemical staining/CD34/NC.jpg]

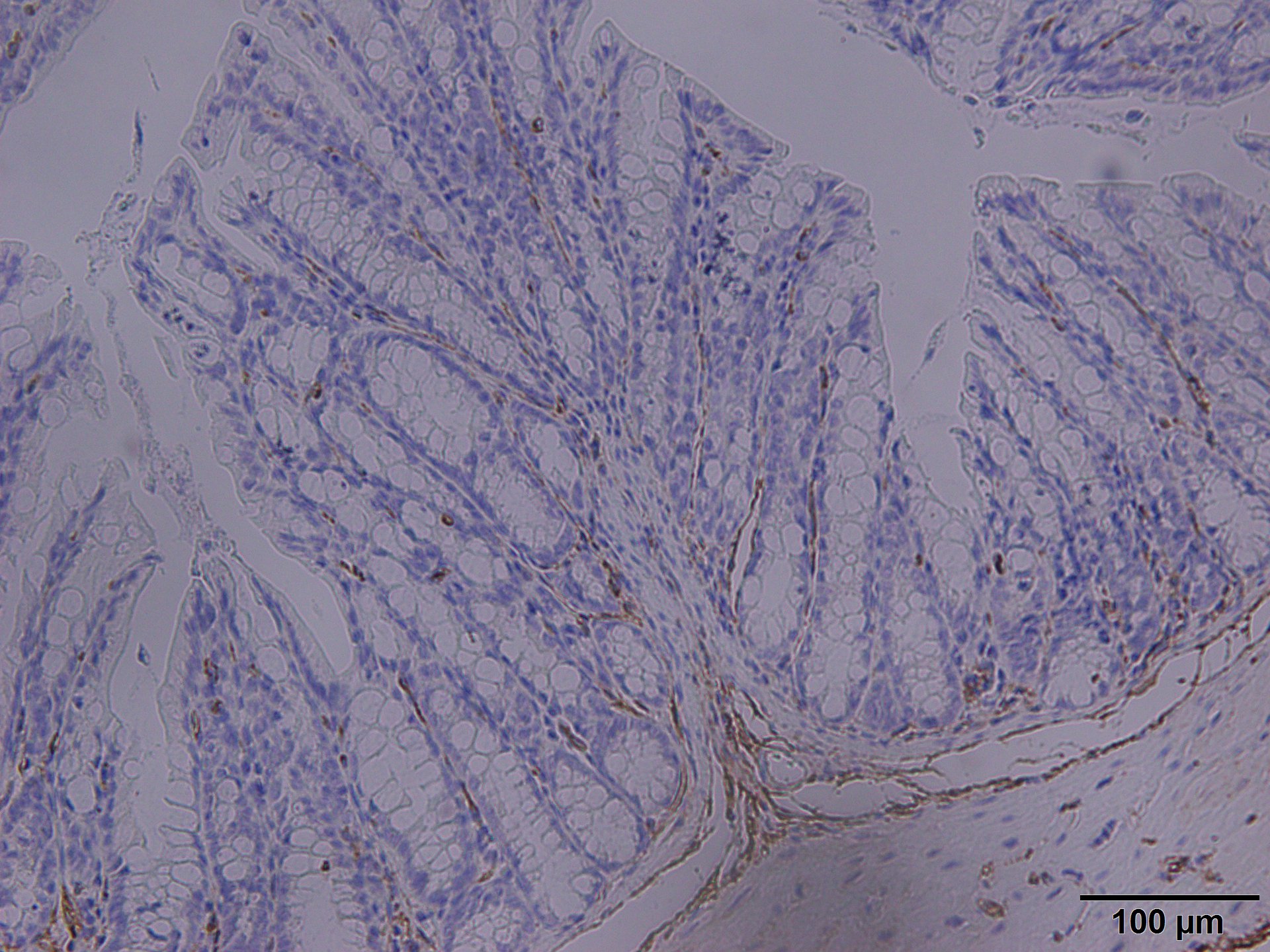

Supplement: Supplementary file 1 [file Data_Sheet_1.ZIP › Raw data/Raw data/Raw data/Figure 6. immunohistochemical staining/CD34/SD.jpg]

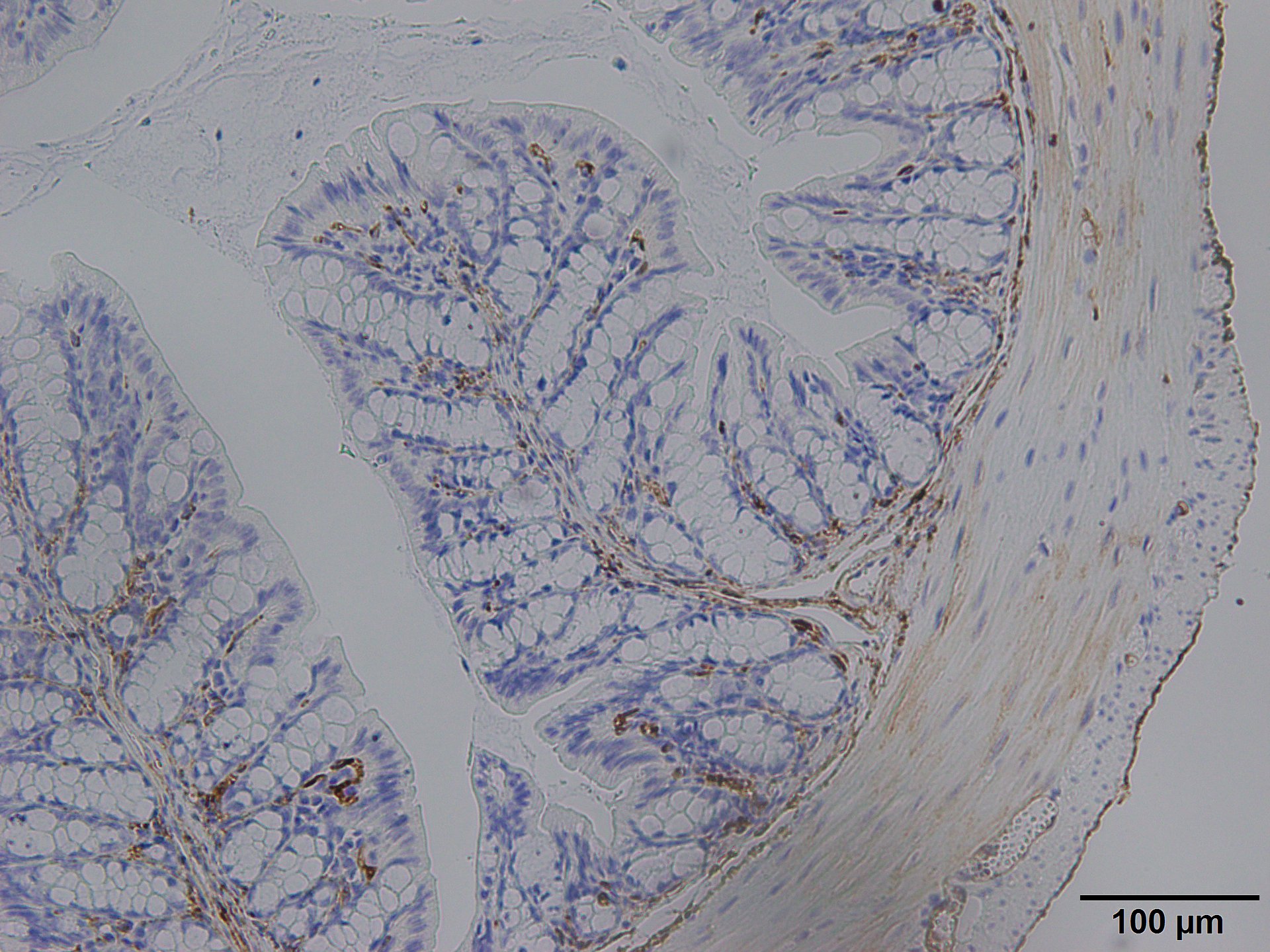

Supplement: Supplementary file 1 [file Data_Sheet_1.ZIP › Raw data/Raw data/Raw data/Figure 6. immunohistochemical staining/CD34/ZS40-H.jpg]

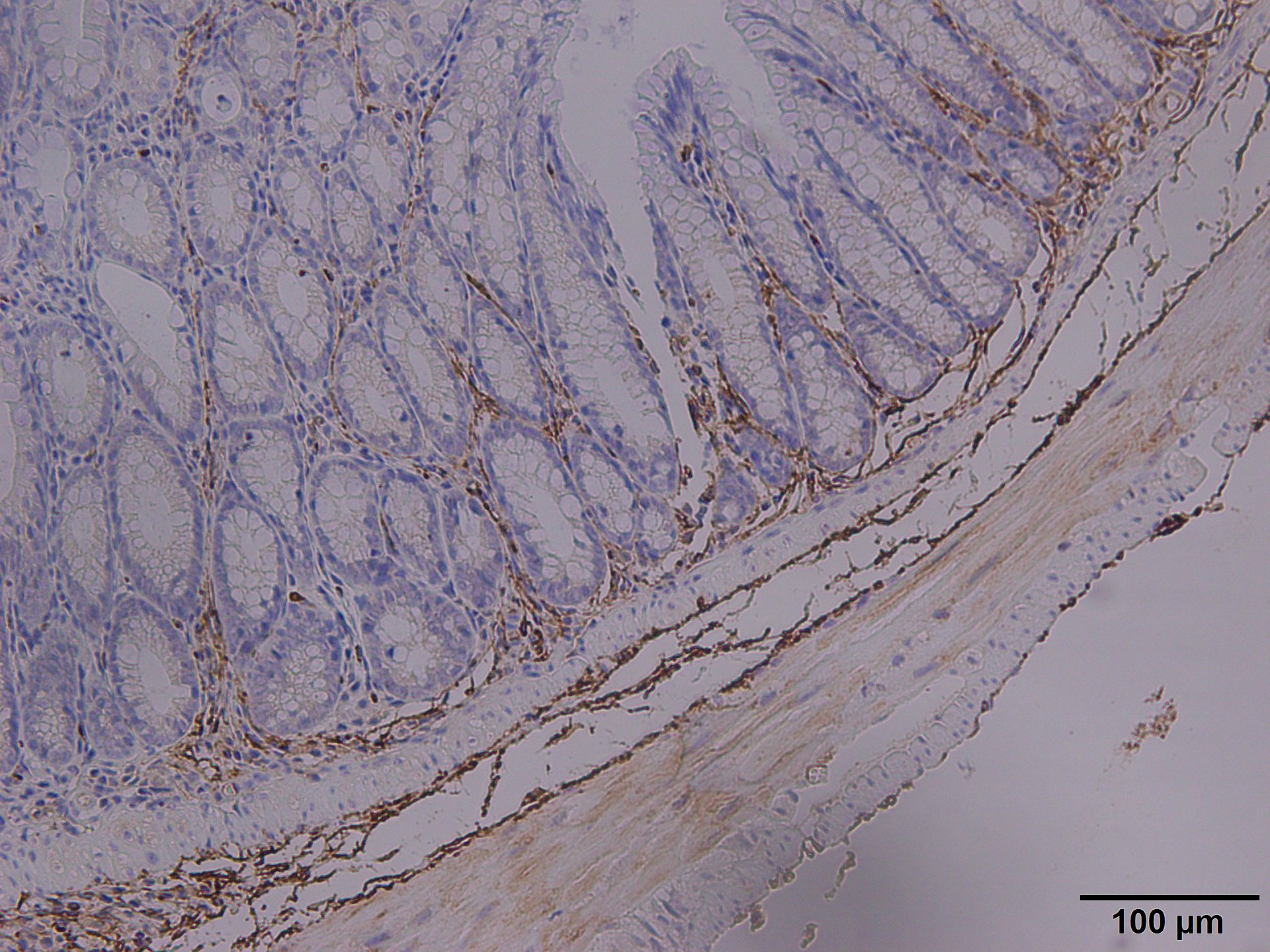

Supplement: Supplementary file 1 [file Data_Sheet_1.ZIP › Raw data/Raw data/Raw data/Figure 6. immunohistochemical staining/CD34/ZS40-L.jpg]

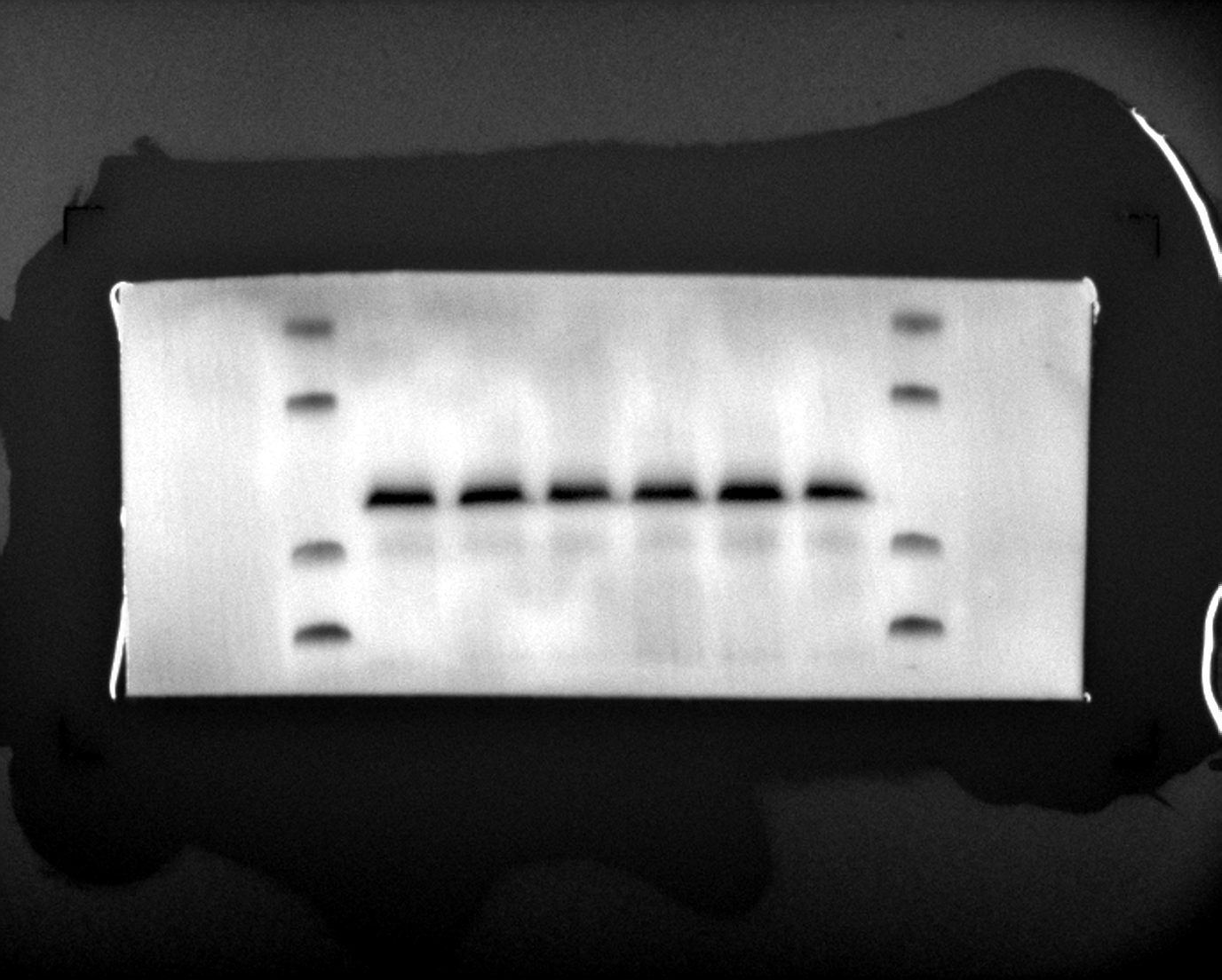

Supplement: Supplementary file 1 [file Data_Sheet_1.ZIP › Raw data/Raw data/Raw data/Figure 8. NFkB-protein/actin.Tif]

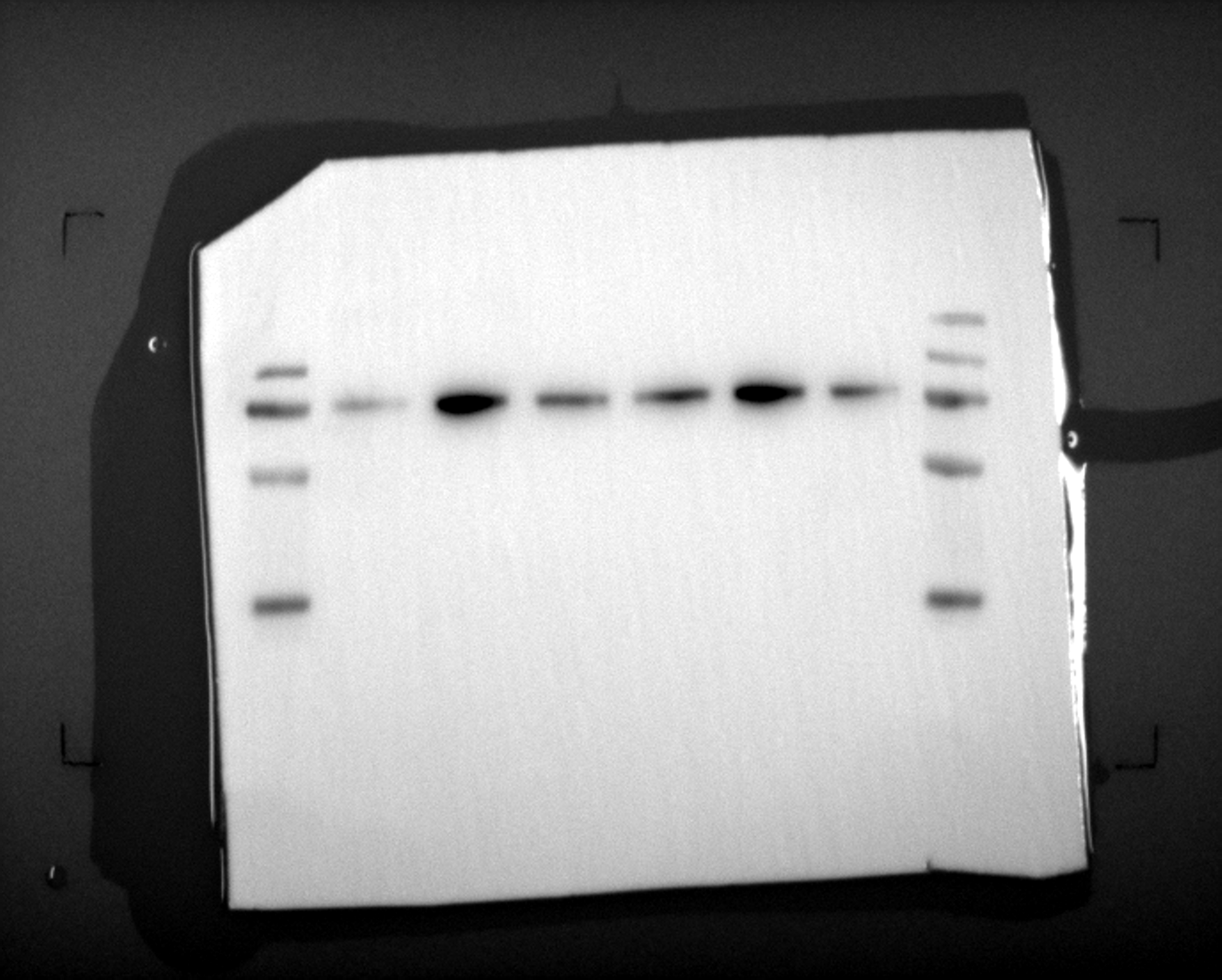

Supplement: Supplementary file 1 [file Data_Sheet_1.ZIP › Raw data/Raw data/Raw data/Figure 8. NFkB-protein/Cox-2.Tif]

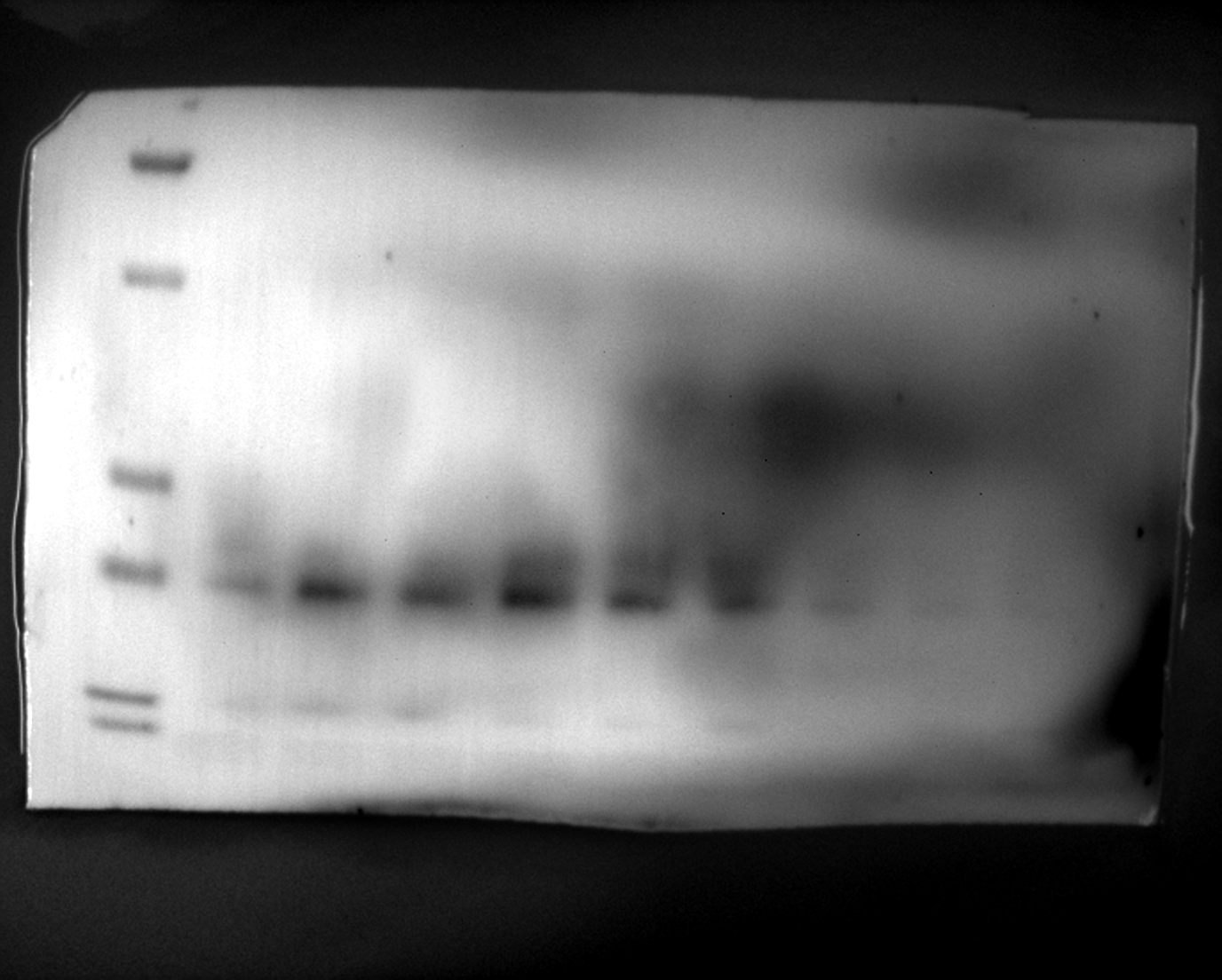

Supplement: Supplementary file 1 [file Data_Sheet_1.ZIP › Raw data/Raw data/Raw data/Figure 8. NFkB-protein/IL-1β.Tif]

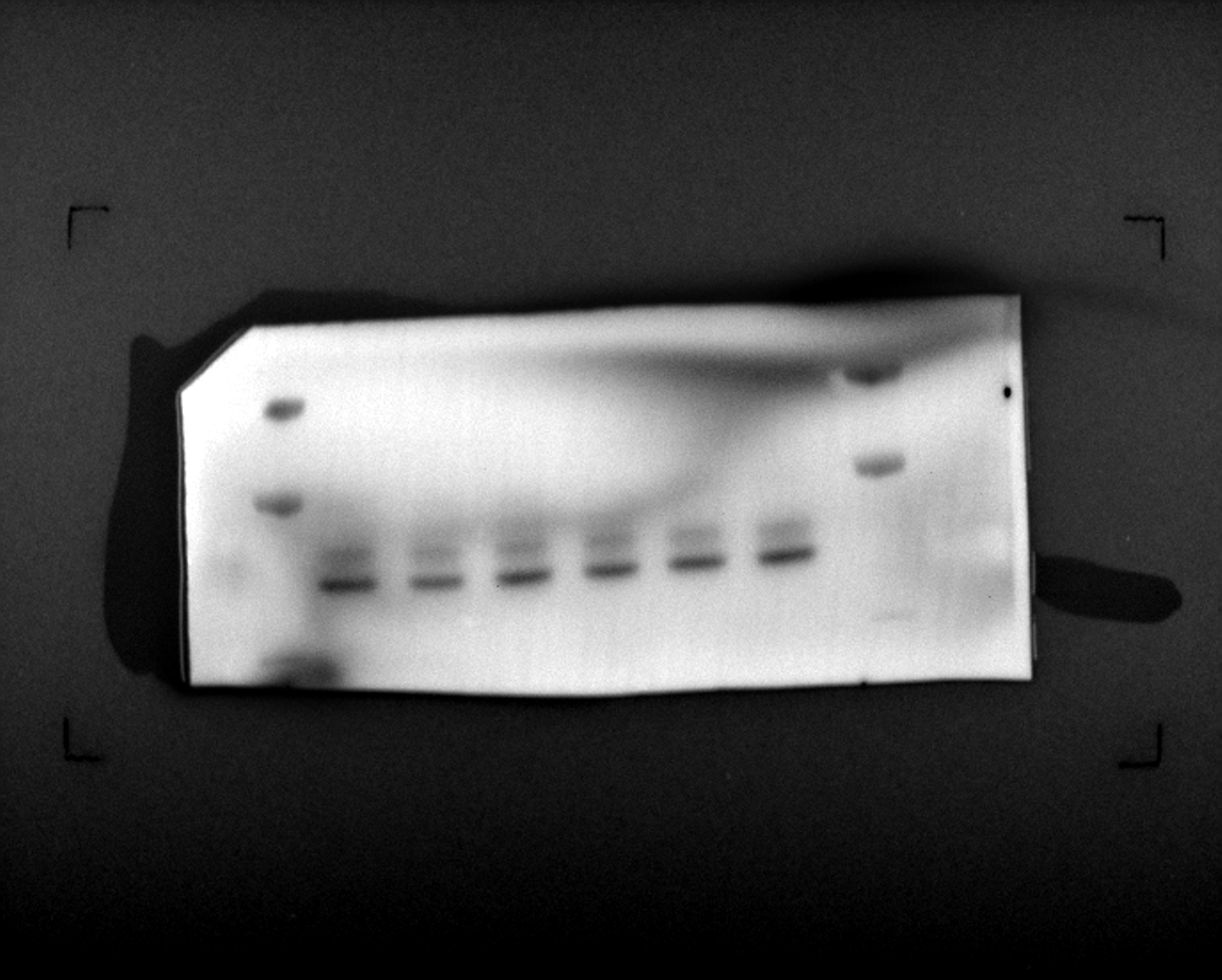

Supplement: Supplementary file 1 [file Data_Sheet_1.ZIP › Raw data/Raw data/Raw data/Figure 8. NFkB-protein/IκBα.Tif]

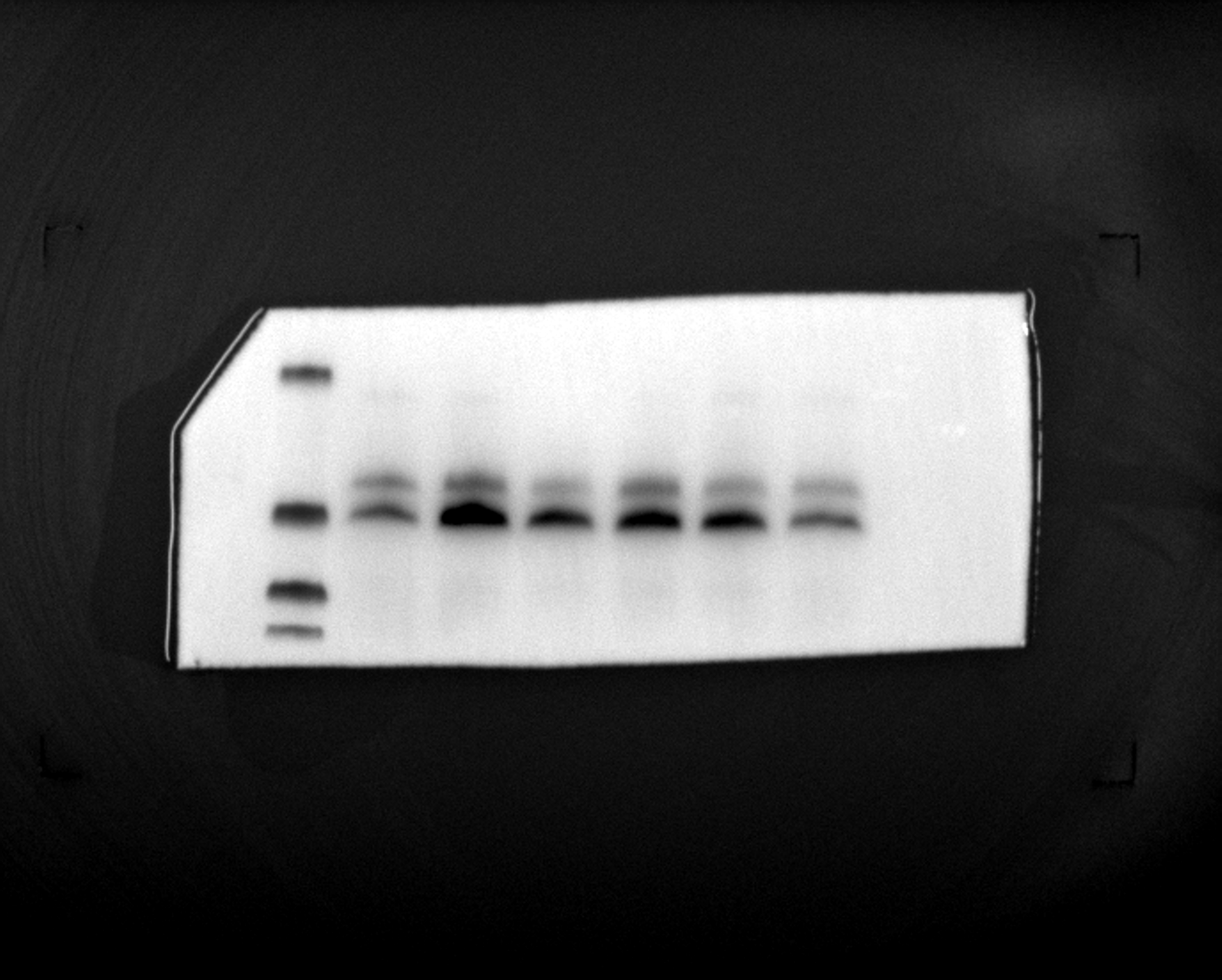

Supplement: Supplementary file 1 [file Data_Sheet_1.ZIP › Raw data/Raw data/Raw data/Figure 8. NFkB-protein/p65.Tif]

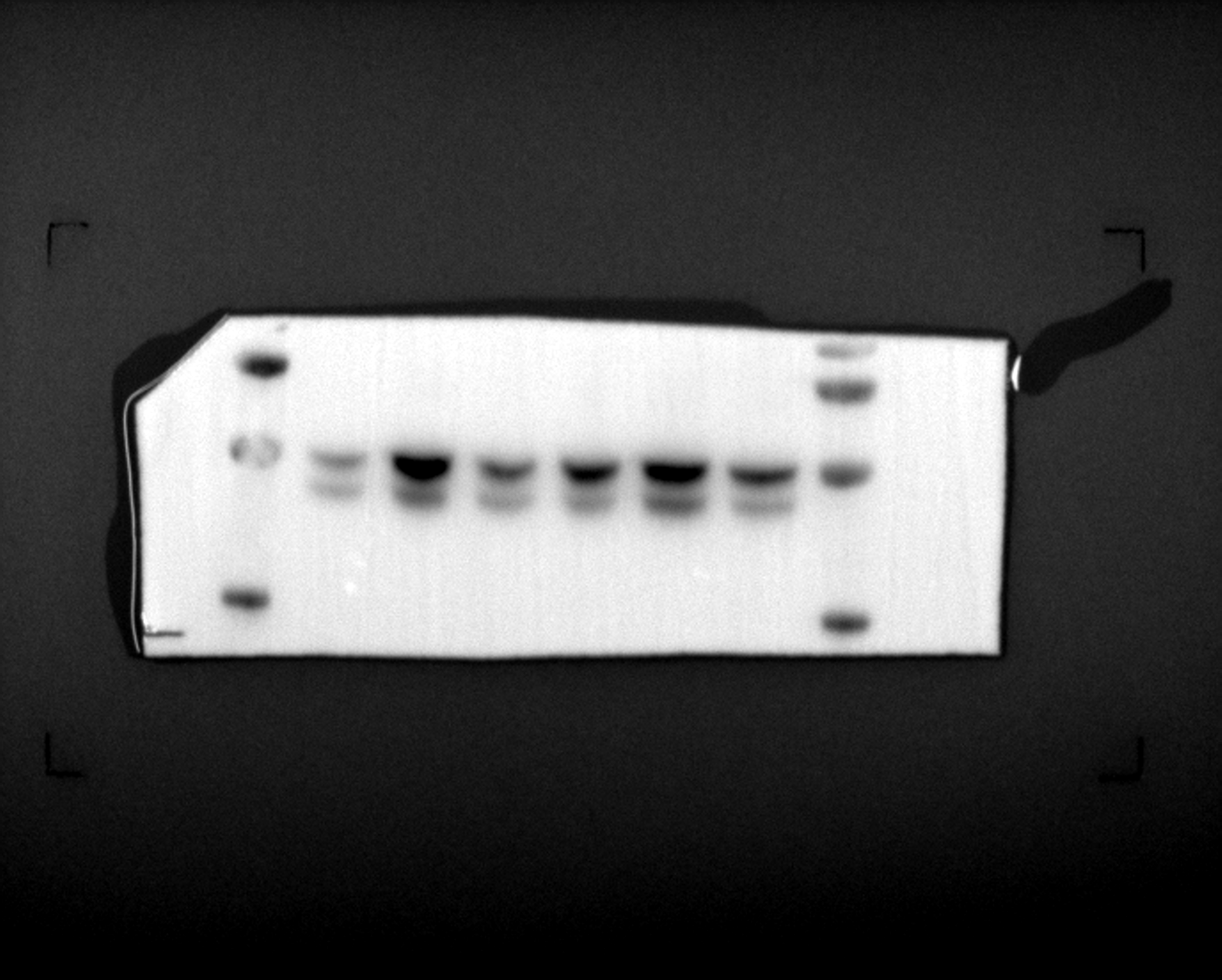

Supplement: Supplementary file 1 [file Data_Sheet_1.ZIP › Raw data/Raw data/Raw data/Figure 8. NFkB-protein/TNF-α.Tif]
